# Oxygen Reduction Reaction Activity in Non-Precious Single-Atom (M–N/C) Catalysts—Contribution of Metal and Carbon/Nitrogen Framework-Based Sites

**DOI:** 10.1021/acscatal.3c00356

**Published:** 2023-05-01

**Authors:** Mengjun Gong, Asad Mehmood, Basit Ali, Kyung-Wan Nam, Anthony Kucernak

**Affiliations:** † Department of Chemistry, Imperial College London, White City Campus, London W12 0BZ, United Kingdom; ‡ Division 3.6—Electrochemical Energy Materials, Bundesanstalt für Materialforschung und -prüfung (BAM), 12203 Berlin, Germany; § Department of Energy and Materials Engineering, Dongguk University, Seoul 04620, Republic of Korea

**Keywords:** fuel cells, single-atom catalysts, oxygen reduction reaction, PGM-free catalysts, M−N/Cs, active site density

## Abstract

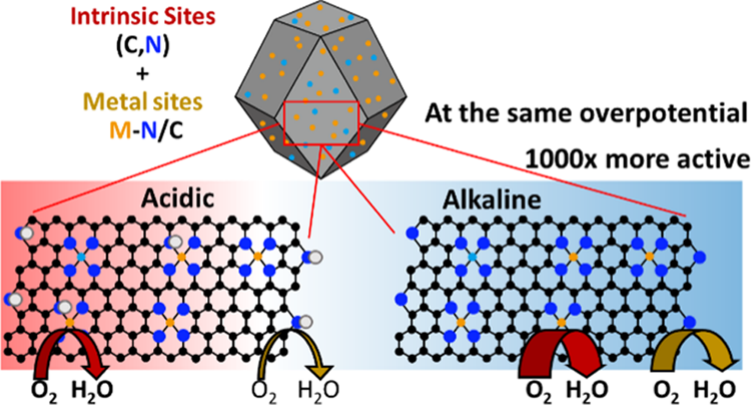

We examine the performance of a number of single-atom M–N/C electrocatalysts with a common structure in order to deconvolute the activity of the framework N/C support from the metal M–N_4_ sites in M–N/Cs. The formation of the N/C framework with coordinating nitrogen sites is performed using zinc as a templating agent. After the formation of the electrically conducting carbon–nitrogen metal-coordinating network, we (trans)metalate with different metals producing a range of different catalysts (Fe–N/C, Co–N/C, Ni–N/C, Sn–N/C, Sb–N/C, and Bi–N/C) without the formation of any metal particles. In these materials, the structure of the carbon/nitrogen framework remains unchanged—only the coordinated metal is substituted. We assess the performance of the subsequent catalysts in acid, near-neutral, and alkaline environments toward the oxygen reduction reaction (ORR) and ascribe and quantify the performance to a combination of metal site activity and activity of the carbon/nitrogen framework. The ORR activity of the carbon/nitrogen framework is about 1000-fold higher in alkaline than it is in acid, suggesting a change in mechanism. At 0.80 V_RHE_, only Fe and Co contribute ORR activity significantly beyond that provided by the carbon/nitrogen framework at all pH values studied. In acid and near-neutral pH values (pH 0.3 and 5.2, respectively), Fe shows a 30-fold improvement and Co shows a 5-fold improvement, whereas in alkaline pH (pH 13), both Fe and Co show a 7-fold improvement beyond the baseline framework activity. The site density of the single metal atom sites is estimated using the nitrite adsorption and stripping method. This method allows us to deconvolute the framework sites and metal-based active sites. The framework site density of catalysts is estimated as 7.8 × 10^18^ sites g^–1^. The metal M−N_4_ site densities in Fe−N/C and Co–N/C are 9.4 × 10^18^ sites^–1^ and 4.8 × 10^18^ sites g^–1^, respectively.

## Introduction

Single atom catalysts (SACs) have become a research frontier in the field of heterogeneous catalysis because of the maximization of atom utilization and the remarkable catalytic performance in both organic^1−5^ and electrochemical processes.^6−9^ For organic synthesis, SACs are widely used in the hydrogenation reaction,^1,10^ oxidation,^2,4,5^ and methane transformation.^3^ On the electrochemical side, SACs for the oxygen reduction reaction (ORR) and the oxygen evolution reaction (OER) are thriving research fields because typical catalysts used for these reactions, especially in acidic electrolytes are based on platinum-group metals (PGMs), which are scarce materials. These reactions are kinetically sluggish and are key steps for fuel cells, electrolyzers, and metal–air batteries.^11−13^ Therefore, non-precious metals like Fe- and Co-based, SACs generally represented as M–N/Cs are being extensively investigated as promising alternatives.^7,11,12,14−19^ In addition to that, Ni, Cu, Fe, and Co SACs have shown good activities for electrochemical carbon dioxide reduction reaction (CO_2_RR).^20−24^ Other interesting applications where SACs have shown promising activities include electrochemical production of hydrogen peroxide,^25−27^ hydrogen evolution reaction (HER),^28−30^ and nitrogen reduction reaction (NRR),^31^ etc. However, relatively poor volumetric activity of SACs precludes them from many applications in industry due to the desire for reaction intensification. In order to improve the catalytic performance, it is important to increase the active site density of SACs and understand how site density influences the overall activity of the catalyst.^32−34^ Although the metal center(s) is presumed to be the active site in many of the reactions mentioned above, the active site density does not equal the amount of metal present in the catalyst because some metal centers are either inactive or buried and inaccessible to the reacting species.^35^ In fact the situation becomes even more complex as the M–N/Cs potentially contain more than one type of active sites. While high ORR performance of metal-free carbon/nitrogen sites (N/Cs) has been well-documented under alkaline conditions, only little has been understood so far with regard to the acidic ORR activity of those N/C framework sites. There are only a few reports on the ORR activity of N/Cs in acidic electrolytes, which were mostly focused on comparing the ORR performance in acidic and alkaline electrolytes.^36−39^ A deconvolution of the site density and activity (turnover frequency TOF) of the N/C framework sites from metal M–N_4_ sites is important to improve our understanding of M–N/C performance patterns in different pH conditions, which still largely remains uncovered. This is possibly due to the difficulties in preparing M–N/C catalysts with the well-defined structure of active sites and a lack of appropriate analytical methods to quantify the density and TOFs of active sites in these types of catalyst materials.

Chemisorption of CO gas at room temperature and electrochemical CO stripping are often used to estimate the active site density for PGM-based catalysts due to the strong bond formed between CO and PGM metals in a crystal lattice, and the ease with which the blocking of catalyst sites by CO adsorption can be linked to the loss in their catalytic activity.^40,41^ However, non-precious M–N/C catalysts, like Fe–N/C, do not show measurable chemisorption of CO at room temperature nor does CO poison the ORR.^35^ Therefore, quantifying active site density by CO chemisorption under ambient conditions is difficult. Strasser and co-workers have developed an *ex situ* measurement for iron active sites in Fe–N/C, the most studied SAC, by CO pulse chemisorption at −80 °C and ^57^Fe Mössbauer spectroscopy techniques.^35,42^ However, as those are *ex situ* techniques to determine the active site density, it becomes difficult to establish a direct correlation between the active site density and the corresponding electrochemical activity of the catalyst.^43^ Many other ionic and gaseous probes including hydrogen sulfide, cyanide, and thiocyanide have also been reported, which interact with active sites in Fe–N/C catalysts strongly but these interactions are mostly irreversible and therefore do not provide accurate quantification of the active sites.^44−48^

In 2016, our group developed an *in situ* method that used NO as a probe molecule, which adsorbed reversibly on active centers in the Fe–N/C catalyst in a liquid electrolyte leading to poisoning of ORR activity, which was recovered in the subsequent step by electrochemical stripping of adsorbed NO. This method offered the advantages of reversible poisoning of Fe–N/C activity and near-realistic measurement conditions (liquid electrolyte pH 5.2).^49^ We also showed that NO can come from a range of different sources including NO (nitric oxide), NO_2_^–^, (nitrite) and NH_2_OH (hydroxylamine).^49^ In a later study, we showed a correspondence between gas-phase chemisorbed NO and adsorption of NO in an electrochemical environment.^50^ For electrochemical experiments, liquid-phase nitrite (NO_2_^–^) is often the preferred poisoning species because it is easy to handle. The nitrite anion interacts with Fe centers strongly, but this interaction, unlike CN^–^ ions, does not irrevocably destroy the catalyst and the catalytic activity can be re-established by electrochemical reduction, which is quite similar to the CO stripping used for Pt catalysts.^51^ We also showed that the same process occurs in the gas phase using NO and that gas-phase poisoning of the catalyst allows a direct link between *ex situ* chemisorption measurements and *in situ* NO poisoning experiments.^50^ Our work for the first time made it possible to estimate the active site density of the Fe–N/C catalyst electrochemically and directly link the loss in performance to the number of sites that were poisoned—allowing an unequivocal linkage between reversible poisoning and performance loss. This approach is easy to operate because no other special equipment is required and gives a reliable active site density (SD) and turnover frequency (TOF).

In this study, we prepared a number of different SACs by a (trans)metalation approach,^16^ which means that different metal centers are coordinated into the same carbon/nitrogen framework.^18^ This approach is unique because the active metal ions are not present during the formation of the carbon/nitrogen scaffold, thus preventing significant morphological changes to the framework during the first pyrolysis step (e.g., changes in surface area, microporosity, amount of nitrogen incorporation, etc).^17,52^ In that way, all the SACs prepared have nearly identical framework morphology and nitrogen functionalities and differ only in the type of active metal centers. The ORR activity of the synthesized SACs is measured in three different pH electrolytes to study the influence of the metal centers and activity trend of the carbon/nitrogen framework with varying pH values. Interestingly, most of the SACs we tested showed very similar ORR activity in the same electrolyte, except for Fe and Co, suggesting that the metal sites were not responsible for the activity, but instead, the performance is associated with the activity of the carbon/nitrogen framework. Moreover, the similarity in the site density of different SACs calculated by nitrite stripping also suggests the existence of two different active sites in these catalysts: framework sites (associated with the carbon/nitrogen (N/C) framework) and M–N_4_ sites. Finally, we show that the nitrite ions not only interact with M–N_4_ metal centers but also with the carbon/nitrogen framework for the tested materials. By comparing a variety of SACs, the density and TOF of framework N/C and M–N_4_ sites are calculated separately.

## Methods

### Preparation of Catalysts

All catalysts were synthesized based on our previously published approach, which involves three main steps.^16^ A commercial ZIF-8 (100% trade name Basolite Z1200), as a source of nitrogen and carbon, was pyrolyzed at 900 °C for 1 h in a tube furnace (Carbolite) under flowing Ar gas (99.99%, BIP plus-X47S, Air products) and the heating rate of the tube furnace was 3 °C min^–1^. The resultant N/C was leached in 2 M H_2_SO_4_ (95%, VWR) overnight at 80 °C to remove Zn, which acted as the templating metal in order to obtain a nitrogen-doped carbon (N/C) framework. For the second step, the non-precious metal ions were (trans)metalated into the N/C framework. 100 mg of N/C was dispersed in 100 mL of methanol (VWR) containing 25 mg of non-precious metal salt including FeCl_2_·4H_2_O (Honey Fluka 99.0%), CoCl_2_·6H_2_O (Fluka Analytical, ≥98.0%), NiCl_2_·6H_2_O (Sigma-Aldrich, ≥99.0%), CuCl_2_·2H_2_O (Sigma-Aldrich, ≥99.0%), MnCl_2_·4H_2_O (Sigma-Aldrich, ≥98.0%), SnCl_4_·5H_2_O (Sigma-Aldrich, 98.0%), SbCl_3_ (Sigma-Aldrich, ≥99.0%), or Bi(NO_3_)_2_·5H_2_O (Sigma-Aldrich, 99.99%). The mixture of N/C and non-precious metal solution was heated under reflux overnight with stirring, followed by thorough washing in deionized water (MilliQ 18.2 MΩ cm) and then overnight washing in 0.5 M H_2_SO_4_ at room temperature to remove the physically adsorbed non-precious metal ions. Afterward, this non-precious metal-coordinated catalyst was subjected to a thorough aqueous washing and was then dried in a vacuum oven overnight. Lastly, a second heat-treatment step was carried out at 900 °C for 1 h in a tube furnace under a flowing 5% H_2_/N_2_ (BOC) gas mixture and the heating rate was 3 °C min^–1^. A reference (Zn)–N/C material was obtained using the same preparative steps but without (trans)metaltion and therefore did not contain any active metal center (except residual Zn from ZIF-8 precursor).

### Physicochemical Characterizations

X-ray photoelectron spectroscopy (XPS) was performed on a Thermo Scientific K-Alpha X-ray photoelectron spectrometer system (Al Kα, 1486.6 eV). All data were recorded at a power of 19.2 W and an X-ray beam size of 400 μm × 200 μm. Survey scans were recorded at a pass energy of 200 eV, and high-resolution scans were recorded at a pass energy of 40 eV. All sample data were recorded at a pressure below 10^–8^ Torr and a room temperature of 294 K. Data were analyzed using CasaXPS software and calibrated with C 1s peak at 284.8 eV. Inductively coupled plasma mass spectrometry (ICP-MS) was performed on an Agilent ICP-MS 7900. Nitrogen physisorption analysis was done to calculate the Brunauer–Emmett–Teller (BET) surface area, pore size, and pore volume by a Micromeritics Tristar II 3020 instrument. The powder catalysts were dried at 140 °C overnight under nitrogen gas before the measurement. High-purity nitrogen gas (BIP plus-X47S) was used during analysis and high-purity helium gas was used for the free-space measurement. Transmission electron microscopy (TEM) was performed using a FEI Titan Tecnai G2 F20 electron microscope. X-ray absorption spectroscopy (XAS) at transition metal K-edges was performed at the 10C beamline of the Pohang Accelerator Laboratory (PAL) in fluorescence mode. As references, XAS of metallic foils and metal phthalocyanines were also measured for energy calibration. X-ray absorption near-edge structure (XANES) and Fourier transform (FT) of the extended X-ray absorption fine structure (EXAFS) spectra were processed using Athena software. The extracted EXAFS signal, χ(*k*), was weighted by *k*^3^ to emphasize the high-energy oscillations and then Fourier transform (FT) occurs in the *k*-range of 3.0–11.0 Å^–1^. Note that the FT magnitude plots of EXAFS spectra are not phase corrected and as such the real bond length should be ∼0.4 Å longer than that shown in the plots. Raman spectra were obtained on a Bruker confocal Raman microscope SENTERRA II. The wavelength used was 532 nm and the substrate was the glass slide.

### Nitrite Stripping

The procedure of nitrite stripping follows that of our previous work.^51^ 5 mg of the catalyst was mixed with 1038.5 μL of isopropanol solution (1:1 volume ratio of IPA/H_2_O) and 54 μL of Nafion (5 wt % solution, Sigma-Aldrich) to prepare the ink and the ink was sonicated for at least 30 min. 8.56 μL of ink, which gave a loading of 0.2 mg cm^–2^, was deposited on the glassy carbon disk of a rotating disk electrode (RDE) with a mirror polished glassy carbon disk and rotator model AFMSRCE. The same ink composition and catalyst loadings were used for all electrochemical measurements (both nitrite stripping and ORR activity measurements in different pH electrolytes) in this study. A custom-made three-compartment electrochemical glass cell was used; 0.5 M acetate buffer at pH 5.2, which is used as the electrolyte was prepared from sodium acetate (99%, Sigma-Aldrich), glacial acetic acid (AnalR Normapur, VWR) and ultrapure water (MilliQ 18.2 MΩ cm). The pH was confirmed using a pH meter (Thermo Scientific, Orion Versastar). Glassy carbon was used as the counter electrode and a saturated calomel electrode (Sentek) was the reference electrode. A potentiostat (Autolab, model PGSTAT20) was used for current and potential control during the electrochemical measurements and the rotating speed was 1600 rpm. Ultrapure nitrogen and oxygen gases (BIP plus-X47S, Air products) were utilized in all experiments. An important aspect of the electrochemical measurements is the requirement of using current integration during the surface electrochemistry (background and stripping scans) of the catalyst. Surface electrochemical processes, such as those occurring on the catalyst, may be incorrectly measured if the common default configuration of staircase voltammetry with sampled current measurements is used.

**Scheme 1:**
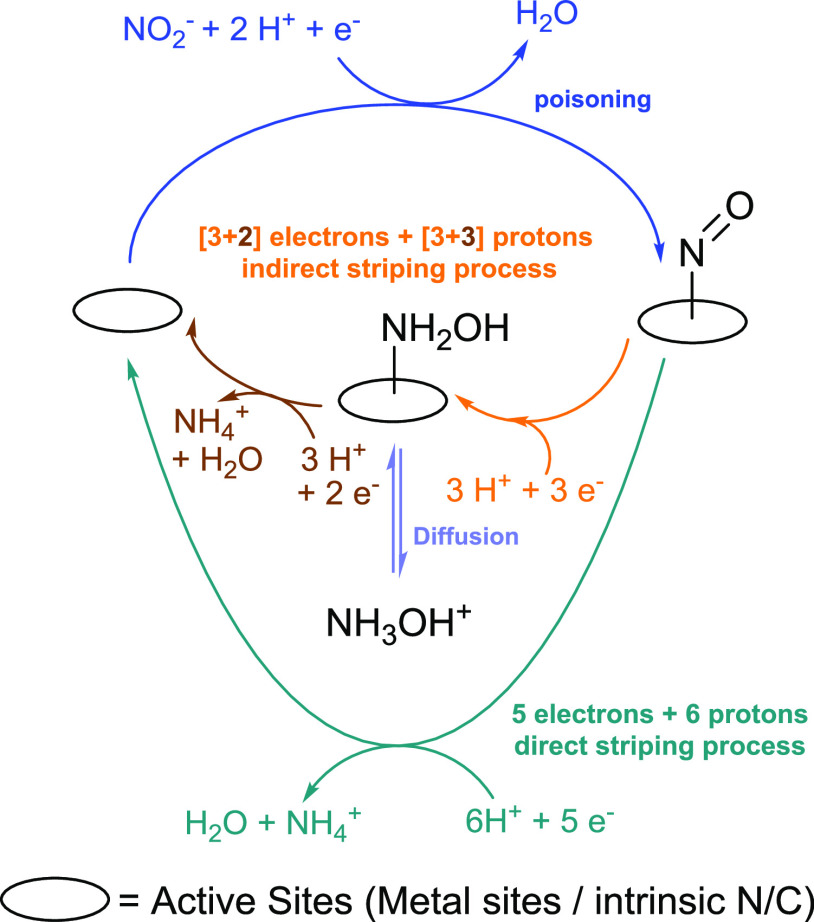
Reaction Scheme for the Adsorption and Reduction of Nitrite Ions at M–N_4_ Active Site

All electrochemical potentials are listed versus the reversible hydrogen electrode (RHE) and the electrolyte was acetate buffer solution. The detailed nitrite stripping methods are shown in Supporting Figure S1. The catalyst was electrochemically cleaned by cycling (2 cycles, with a scan rate 5 mV s^–1^ between 1.00 and 0.00 V_RHE_) in O_2_-saturated electrolyte and activated under N_2_-saturated electrolyte with 10 cycles of scanning between 1.10 and −0.3 V_RHE_ with 10 mV s^–1^ and 5 cycles with 1 mV s^–1^. Then, the cleaning step was repeated under O_2_ and N_2_ again. The measuring step involved three electrochemical measurements: (1) oxygen reduction reaction performance (2 cycles, 5 mV s^–1^, between 1.00 and 0.30 V_RHE_); (2) pre-baseline under nitrogen (2 cycles, 10 mV s^–1^, between 1.00 and 0.30 V_RHE_); and (3) baseline under nitrogen (2 cycles, 10 mV s^–1^, between 0.50 and −0.20 V_RHE_). For all these three steps, the electrode was rotated at 1600 rpm. The nitrogen pre-baseline data provide the background scan for correcting the ORR data whereas the nitrogen baseline scan provides the background scan for the nitrite stripping step. Then the catalyst was poisoned by a four-step process using a rotation rate of 300 rpm and during the poisoning step, the electrode was at the open circuit potential (OCP). The catalyst was poisoned by immersing the electrode into 0.125 M NaNO_2_ solution for 5 min, which allowed the nitrite ions to form a bond with metal as shown in the first reaction of Scheme 1. Then, 1 min of water washing was introduced to remove the excess nitrite ions, which were physically adsorbed on the surface of the catalyst. Then the catalyst was immersed in acetate buffer solution (pH = 5.2) to stabilize the NO, which is bonded with the catalyst. Malko et al. suggested that the nitrite should interact with the metal center as a nitrosyl ligand after immersing in acidic solution.^49^ Another 1 min of water washing was done to complete the poisoning step. After poisoning, the measuring step as described above was repeated including ORR, pre-baseline, and baseline. The poisoned ORR was recorded and then nitrogen cyclic voltammetry (CV), including the pre-baseline and baseline measurements. In the baseline measurement (between 0.5 and −0.2 V_RHE_), NO was reduced via a five-electron reaction as shown in step 2 of Scheme 1, and the ORR performance of the catalyst was recovered after this reduction step. Therefore, the recovery measurements were done to confirm that the catalytic performance had been re-established. In order to calculate the stripping charge more accurately, a second time poisoning was done and the poisoning step was exactly the same as the first time. However, after the second poisoning, ORR performance was not recorded; only the nitrogen CVs were taken. Based on the baseline region of nitrogen CV, the stripping charge (*Q*_strip_) could be calculated by eq 1.
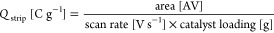
1The area is calculated by the difference in baseline nitrogen CV between poisoned and recovery scans (see the “Nitrite Stripping to Quantify Active Sites in Different M–N/C Catalysts” section). The stripping charge was used to calculate the site density (SD) as shown in eq 2.
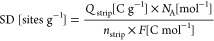
2*F* stands for the Faraday constant and *n*_strip_ is the number of electrons involved in the reduction of one nitrosyl adsorbed on the metal centers. The value of *n*_strip_ reported for the reduction step can vary between 3 and 5 leading to different final products such as hydroxylamine or ammonium ion as shown in Scheme 1.^53^ As we reported previously, ammonium ions are more likely to be produced. Therefore, we use a *n*_strip_ value of 5.^49,51^

**Scheme 2:**
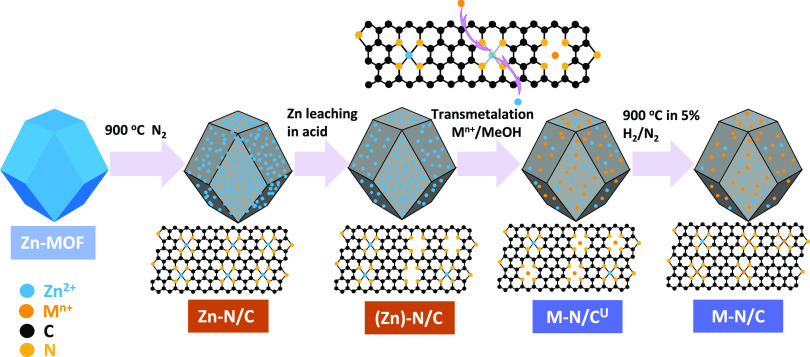
Synthesis of the M–N/C Catalyst via (trans)Metalation U stands for the unactivated catalyst, which means the catalyst did not undergo second pyrolysis under 5% of H_2_/N_2_ i.e., no activation step.

Once the site density is calculated, the turnover frequency (TOF) could be calculated as shown in eq 3, where Δ*i_k_* is the change in the kinetic current between poisoned and unpoisoned ORR.
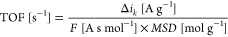
3

### Oxygen Reduction Reaction

Different M–N/C catalyst inks with a loading of 0.2 mg cm^–2^ were deposited on the glassy carbon disk of a rotating ring-disk electrode (RRDE, Pine Instruments, model AFE6R1AU with glassy carbon as a disk with a concentric gold ring and rotator model AFMSRCE). A custom-made three-compartment electrochemical glass cell was filled with either 0.5 M sulfuric acid (VWR, 95.0%) or 0.1 M KOH (VWR, 85.3%) electrolytes. A reversible hydrogen electrode (RHE) was used as the reference electrode, which was connected to the electrochemical cell via a Luggin–Haber capillary, and a glassy carbon rod was used as the counter electrode. Ultrapure oxygen and nitrogen gases (BIP plus-X47S, Air products) were used in the measurements, which were taken in the potentiostat (Autolab, model PGSTAT20) and all measurements were taken at a rotation speed of 1600 rpm. The dried catalyst was first activated in the O_2_-saturated electrolyte by cyclic voltammetry (CV) between 0.00 and 1.00 V_RHE_ with a scan rate of 10 mV s^–1^ and the CVs for the first three cycles are shown in Supporting Figure S2. After 3 CV cycles, the catalysts were fully activated, and the ORR performance was stabilized. Then the ORR polarization curves were recorded by linear sweep voltammetry (LSV) from 1.00 to 0.00 V_RHE_ with a scan rate of 5 mV s^–1^, and the gold ring current was recorded at an applied potential of 1.50 V_RHE_ to calculate the yield of H_2_O_2_ by eq 4. A high ring potential value was used to fully oxidize the hydrogen peroxide produced for its accurate quantification.
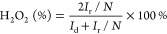
4where *I*_r_ is the current recorded at the gold ring, *I*_d_ is the disk current, and *N* stands for the ring collection efficiency of the RRDE. Subsequently, the nitrogen background was measured by LSV under a N_2_-saturated electrolyte with the same potential range and scan rate as the ORR. Ohmic resistances were determined by electrochemical impedance spectroscopy (EIS) for the iR composition.

### Use of Nitrite Stripping to Characterize Fe–N/C Catalysts Produced Using Different Synthetic Approaches

**Figure 1:**
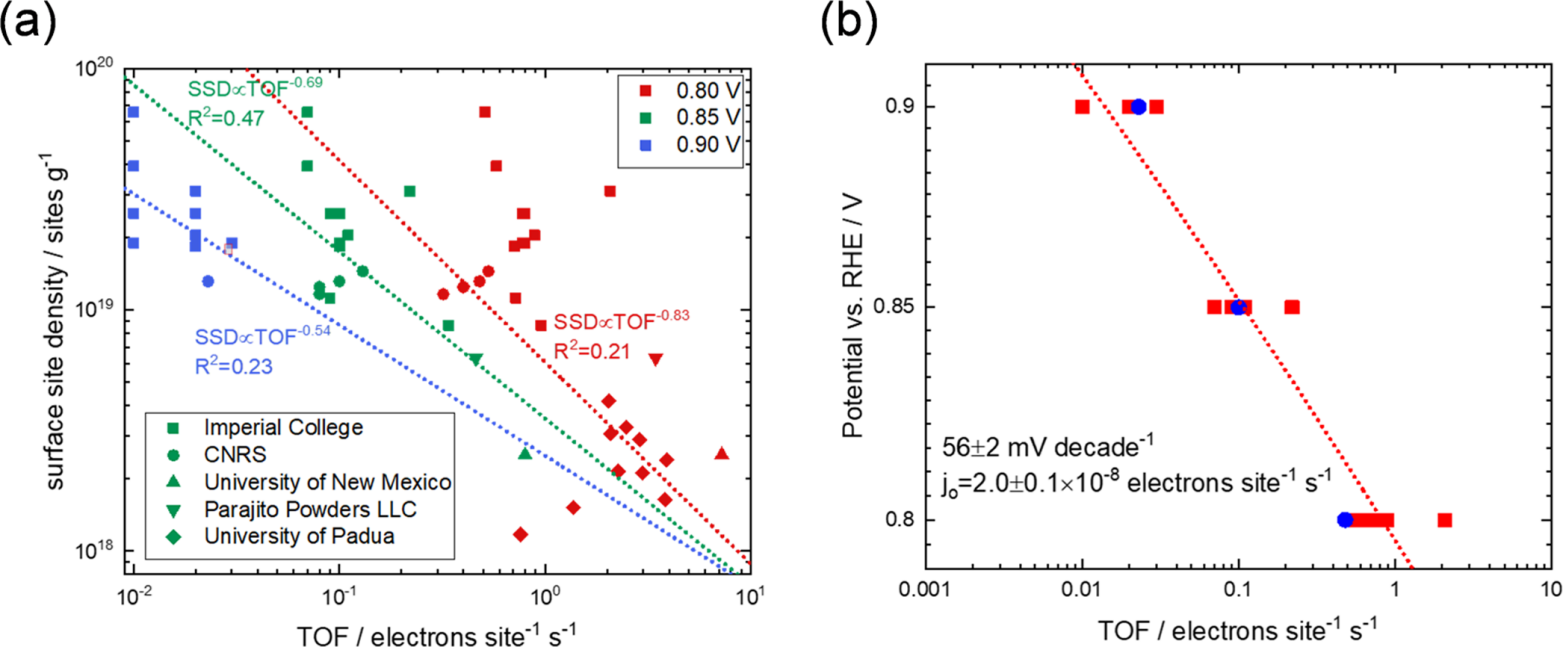
Meta-analysis of site density and TOF of Fe–N/C catalysts produced through different chemistries: (a) Master plot of site density/TOF data for 38 catalysts produced in different laboratories and tested in our laboratory using the nitrite stripping method, data published in refs (32, 54, 55) and this paper and (b) analysis of the Tafel slope and exchange current density of 10 best catalysts (i.e., those which show activity at 0.90 V_RHE_),

In order to compare the nitrite stripping process, we have performed a meta-analysis of the performance of 38 different catalysts^32,54,55^ produced in a variety of different laboratories and tested in our laboratory using the nitrite stripping method as shown in Figure 1a. The performance of the catalysts is determined at 0.80, 0.85, and 0.90 V_RHE_. Although there has been some suggestion in the literature that site density and turnover frequency are inversely related, we do not see any statistically significant relationship between site density and TOF assuming a power expression as shown in eq 5
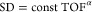
5when α = −1, SD and TOF are inversely related, although this equation uses α as a fitting parameter in order to find the best value that can fit the data. Note that the best-fit lines on the plots show very poor correlation coefficients (<0.5) and the respective exponents of the best-fit curves are >−1, implying that there is no statistically significant correlation and the hypothesis is invalid. It is perhaps intriguing that we do not see a common turnover frequency for all catalysts as might be expected if there was only one active site. Surprisingly, however, if we only concentrate on the most active catalysts, i.e., those which achieve performance at 0.90 V_RHE_ (composed of two different classes of catalyst), we see a close grouping of performance, all with a common Tafel slope (56 ± 2 mV decade^–1^) and exchange current density ((2.0 ± 0.1) × 10^–8^ electrons site^–1^ s^–1^), suggesting that a common site prevails in these catalysts. One interpretation, which we will explore in this paper, is that the wide variation of results seen in Figure 1a is due to the presence of two (or more) sites, which are counted by the nitrite method, but which have different activities. Thus, although we can count the number of sites, our TOF is a composite performance made up of the TOFs of different sites weighted by their proportion.

## Results and Discussion

### Structural Characterization of the M–N/C Catalyst

All M–N/C catalysts are prepared by exchanging Zn with other active metals (M) in a methanol solution and the synthetic steps are shown in Scheme 2 and details are provided in our recent work.^16^ Pyrolyzed zeolitic imidazolate framework-8 (ZIF-8) has been used as the Zn–N/C template and zinc has been removed by acid leaching to generate abundant vacant N/C sites. By refluxing the N/C framework with metal chloride solutions in methanol, active metal centers are effectively coordinated into the vacant N/C sites (via metalation). In parallel, some metal ions will exchange with residual zinc ions (via transmetalation) present in N/C, which have not been removed by acid leaching. However, there may still be some small amounts of zinc left in the final catalyst probably as inaccessible Zn–N*_x_* sites. As we reported in our recent study, there was some nanoscopic iron oxide formed during the reflux step, and was eventually converted into Fe–N_4_ sites upon a second high-temperature pyrolysis step.^16^ Therefore, the catalysts were pyrolyzed at 900 °C again to completely convert any nanoscopic metal oxides into M–N_4_ moieties. The benefit of this method is that all of the catalysts have only minimal differences (if any) in the N/C framework properties in the final materials, which allows us to investigate the role of different metal centers hosted in a nearly identical N/C scaffold.

**Figure 2:**
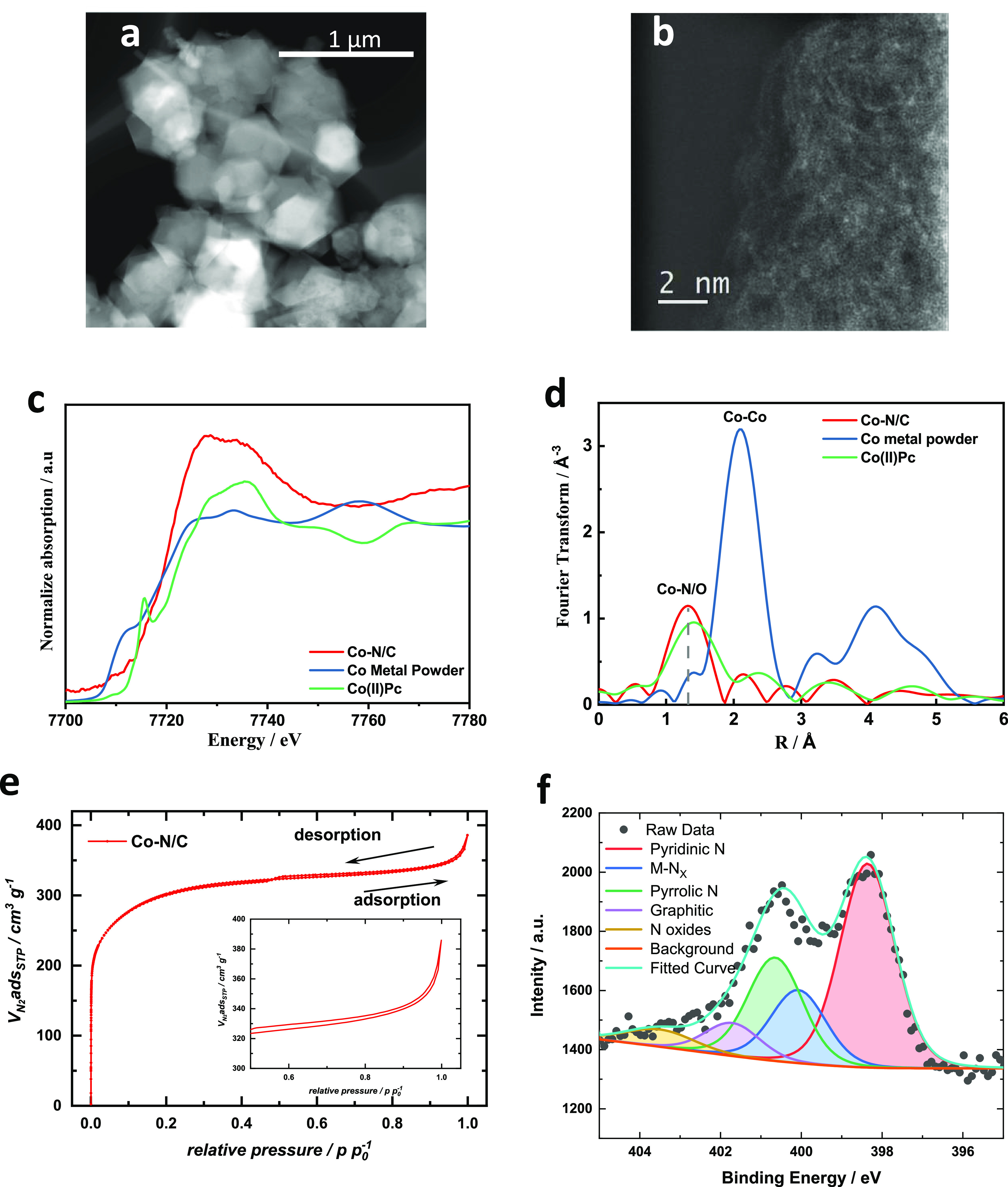
Structural analysis of Co–N/C by (a) TEM; (b) FFT filtered atomic resolution HAADF-STEM image of Co–N/C; (c) Co K-edge X-ray absorption near-edge spectra (XANES); (d) Co K-edge extended X-ray absorption fine structure (EXAFS) profiles of Co–N/C; (e) N_2_ adsorption and desorption isotherm, inset: zoom-in of relative pressure between 0.5 and 1.0; and (f) High-resolution nitrogen 1s X-ray photoelectron spectrum.

Transmission electron microscopy (TEM) images (Figure 2a and Supporting Figures S3–S7) illustrate that the Co–N/C catalyst, which is used as a representative example for all M–N/Cs prepared in this study, has a polyhedron particle morphology closely resembling that of the ZIF-8 precursor. Also, the particle size for M–N/C catalysts is similar with ZIF-8 and is around 400 nm. Figure 2b is a high-angle annular dark-field scanning transmission electron microscopy (HAADF-STEM) image of Co–N/C and is clearly showing that Co is atomically dispersed as bright spots in the amorphous carbon without any visible cluster/nanoparticle formation. Electron energy loss spectroscopy (EELS) analysis was also performed to detect Co and results are shown in Supporting Figure S8. Elemental mapping of different metals in the synthesized M–N/Cs (Supporting Figures S3–S7 and S9) indicates that the metals are evenly distributed. In some cases, the distribution of metal centers in the elemental mapping images is not so evident because of low metal contents. Detailed microscopy imaging and EDS mapping Fe–N/C are shown in Supporting Figure S9 and in our recent work.^16^ Inductively coupled plasma mass spectroscopy (ICP-MS) was used to determine the total contents of active metals in each M–N/C and results are provided in Table 1. For Ni–N/C, Mn–N/C, and Bi–N/C, the active metal loading is relatively low (<0.50 wt %), while for Co–N/C, Sn–N/C, Sb–N/C, and Fe–N/C, the active metal loadings are in the range of 0.50–7.10 wt %.

**Table 1:** Total Site Density (M−N_4_ + N/C sites), ICP-MS Metal Content, Site Utilization, and TOF_total_ of M–N/C Catalysts

catalyst	ICP(metal)/wt %	site density/10^19^ sites g^–1^	metal site utilization/%	TOF_total_ (0.75 V_RHE_)
(Zn)–N/C	8.50	0.30	0.38	0.83
Fe–N/C	7.10	1.42	1.85	3.34
Co–N/C	0.54	0.97	17.5	0.79
Ni–N/C	0.13	0.51	38.5	0.67
Mn–N/C	0.18	0.62	31.59	0.37
Sn–N/C	0.83	0.37	8.67	0.61
Sb–N/C	3.14	0.62	4.01	0.24
Bi–N/C	0.02	0.62	1083^a^	0.16

a This abnormality in calculation of metal site utilization for Bi–N/C is due to low ICP metal content but high site density. It also indicates that the nitrite stripping is likely happening at N/C framework sites.

**Figure 3:**
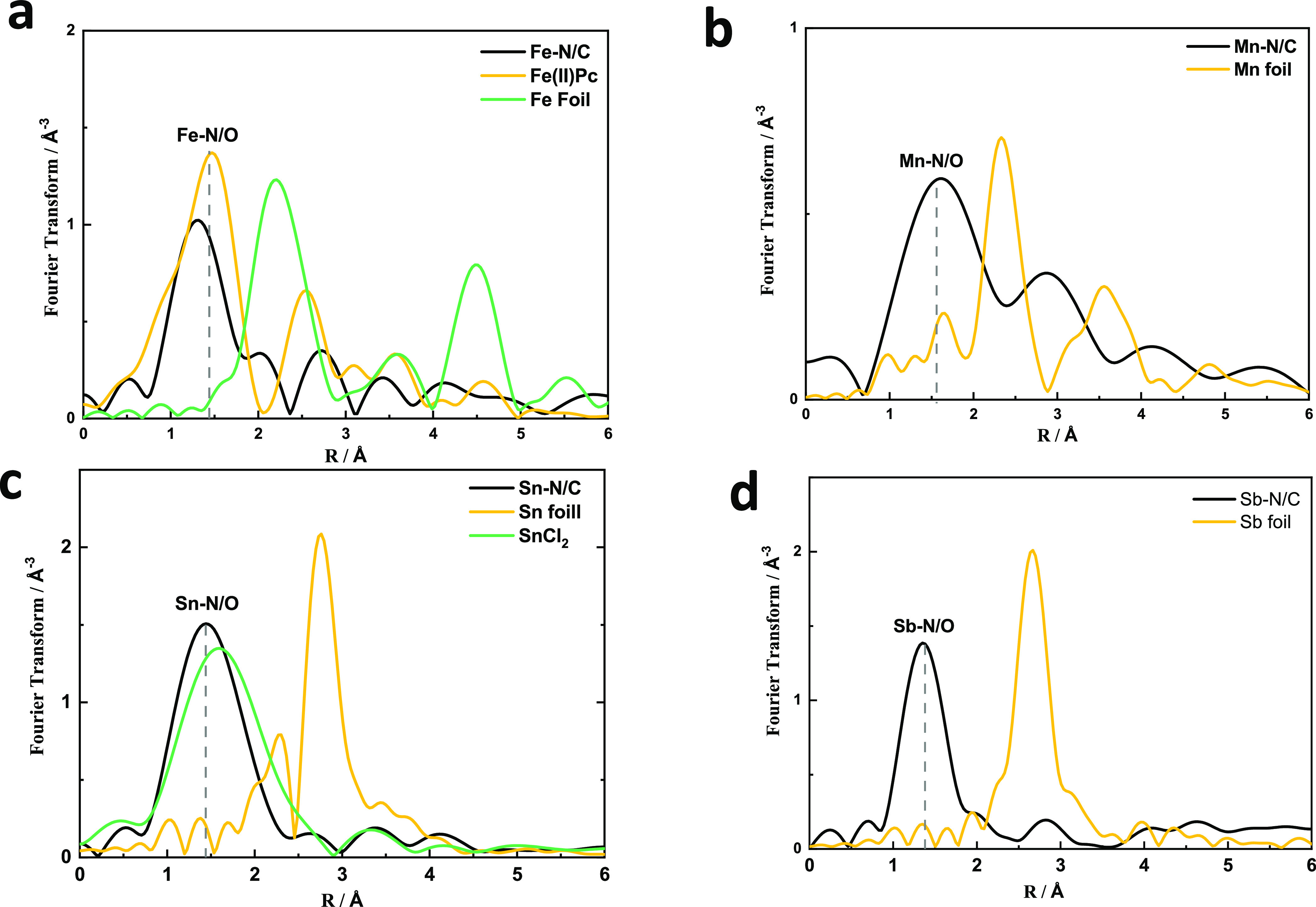
K-edge EXAFS profiles of different M–N/Cs. (a) Fe–N/C; (b) Mn–N/C; (c) Sn–N/C, and (d) Sb–N/C.

X-ray absorption spectroscopy (XAS) measurements were performed on various M–N/C catalysts to analyze the coordination environments of active metal centers in those materials. As can be seen in Figure 2d, the EXAFS spectrum of the Co–N/C catalyst resembles closely that of the Co(II) phthalocyanine [Co(II)Pc] with the main peak centered at around 1.4 Å corresponding to a Co–N/O coordination. In Co K-edge XANES spectrum (Figure 2c), the edge energy (∼7730 eV) suggests that the valence state of Co in Co–N/C is 2+ in accordance with the literature.^56,57^ For the pre-edge region (7708–7710 eV), the Co–N/C has higher intensity compared with Co(II)Pc, which suggests that Co potentially formed non-planar Co–N*_x_*.^56^ The XANES of other M–N/C was shown in Supporting Figure S10. As we have reported in our recent work, the EXAFS spectrum of Fe–N/C fitted well with a coordination number of 4 indicating the formation of Fe–N_4_ sites (see Supporting Figure S11b and Supporting Table S1).^16^ Similarly, the EXAFS spectra of other M–N/C catalysts in Figure 3 show a main peak around 1.4 Å that is generally attributed to a M–N/O coordination.^1,56^ From the EXAFS analysis, it is clear that the M–N/C catalysts that we have prepared using (trans)metalation of a pre-formed N/C framework mainly consist of M–N_4_ sites without any detectable side phases such as metal nanoparticles or metal carbides, etc.

Nitrogen adsorption–desorption isotherms are type I for all samples (Figure 2e and Supporting Figure S12). The Brunauer–Emmett–Teller (BET) surface areas for all the catalysts are in the range of 1000–1150 m^2^ g^–1^ and the micropore volumes are between 0.36 and 0.40 cm^3^ g^–1^. Detailed values of surface areas and pore volumes of all catalysts are provided in Supporting Table S2. The low variability of surface area and pore volume between different samples indicates that the N/C framework mainly retained its morphology in the final materials, which is beneficial for a fair comparison of framework electrocatalytic activities of different metal centers with minimal interference of morphological effects. The XPS analysis of M–N/Cs is shown in Supporting Figures S13 and S14 and the elemental composition analysis based on XPS for all catalysts are shown in Supporting Table S3. The deconvoluted high-resolution N 1s spectra (Figure 2f and Supporting Figures S13 and S14) indicate five different bonding configurations of N atoms, including pyridinic N (398.3 eV), M–N (399.7 eV), pyrrolic N (400.6 eV), graphitic N (401.7 eV), and N oxides (403.5 eV).^16^ A summary of the absolute total N and different N sites in different catalysts is shown in Supporting Figure S15 and Supporting Table S4. The majority of the nitrogen sites (up to 90%) in all catalysts are present as pyridinic N, M–N, and pyrrolic N. The amount sof graphitic N and N oxides are minor. As shown in Supporting Figure S15, a relatively higher pyridinic N fraction is present before the second pyrolysis step as compared to the final activated materials. Raman spectrum of the Fe–N/C catalyst is used to analyze the graphene domain size L_a_ (shown in Supporting Figure S16). The calculated value of L_a_ is 7.3 ± 2.3 nm (average value calculated based on 10 positions of Raman spectra). Based on the calculated value of average graphene domain size, the percentages of basal, edge, and buried Fe–N_4_ sites were estimated in our recent work and shown in Supporting Table S5.^16^

### Oxygen Reduction Activity in Acidic, Near-Neutral, and Alkaline Electrolytes

**Figure 4:**
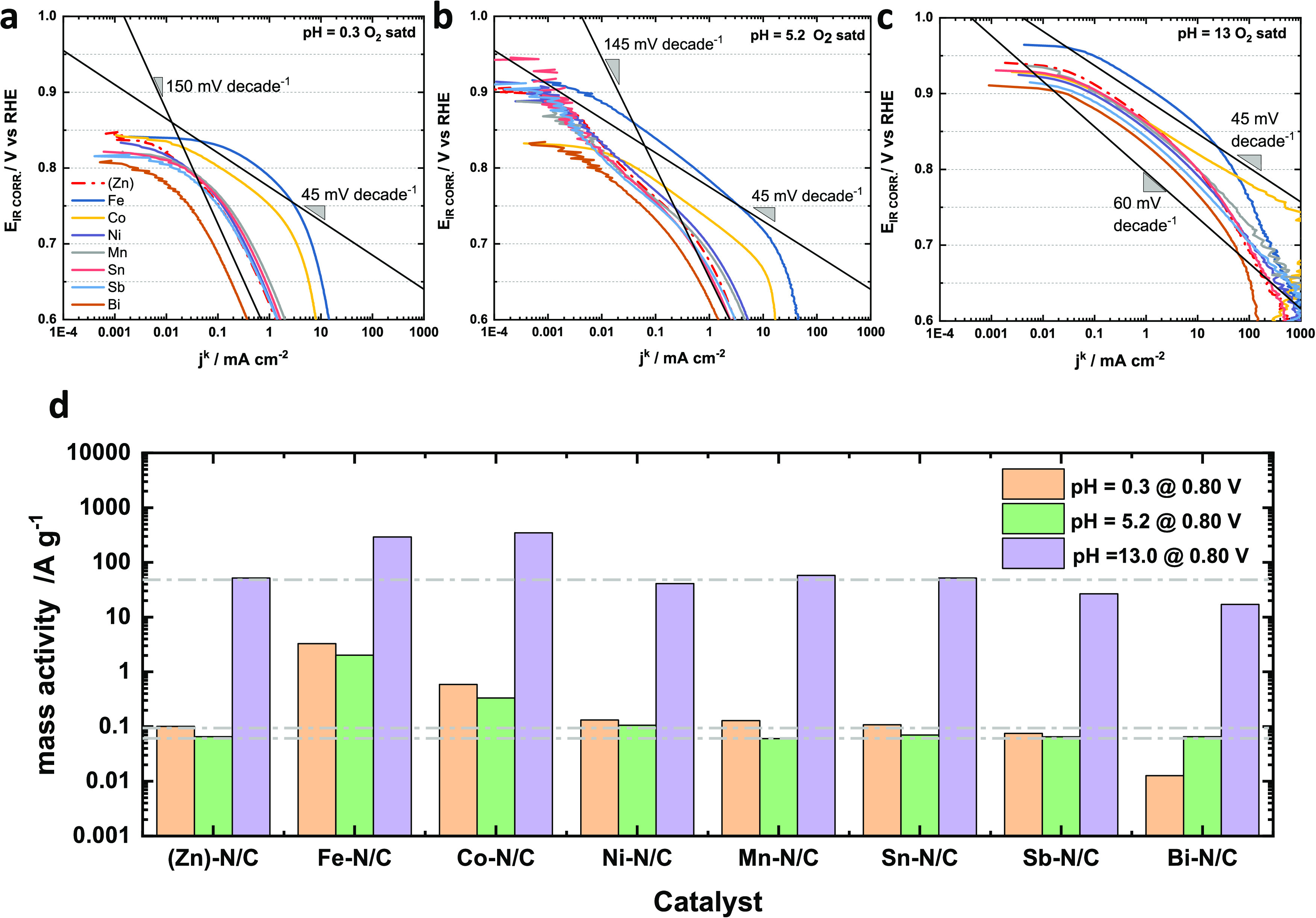
Tafel plot and ORR mass activity comparison for M–N/Cs in different pH electrolytes. (a) Tafel plot of ORR measured in O_2_-saturated 0.5 M H_2_SO_4_ (pH = 0.3); (b) Tafel plot of ORR measured in O_2_-saturated 0.5 M acetate buffer solution (pH = 5.2); (c) Tafel plot of ORR measured in O_2_-saturated 0.1 M KOH (pH = 13.0); (d) Comparison of mass activity in different electrolytes at 0.80 V_RHE_. For all measurements, the scan rate was 5 mVs^–1^ with a rotation speed of 1,600 rpm and a catalyst loading of 0.2 mg cm^–2^.

The ORR performance of all catalysts was tested in acidic and alkaline electrolytes in a rotating ring-disk electrode (RRDE) setup. The ORR linear sweep voltammetry (LSV) responses and hydrogen peroxide yields are displayed in Supporting Figure S17. ORR activity of reference (Zn)–N/C material was also tested and compared to the other M–N/Cs. For all catalysts, the ORR activity improved after the activation step, for example, the half-wave potential (*E*_1/2_) of Co–N/C increased from 0.48 to 0.70 V_RHE_. The ORR performances of all catalysts in acidic (0.5 M sulfuric acid pH = 0.3), near-neutral (0.5 M sodium acetate buffer solution pH = 5.2), and alkaline (0.1 M potassium hydroxide pH = 13) electrolytes were recorded and the detailed ORR plots and %H_2_O_2_ yield are shown in Supporting Figures S17–S24. All catalysts showed a better ORR performance in an alkaline medium than in an acidic medium. In alkaline conditions, most of the catalysts already reached their limiting currents at 0.80 V_RHE_, but in acidic conditions, they barely showed any catalytic performance at 0.80 V_RHE_ with the only exception of Fe–N/C. Moreover, the %H_2_O_2_ yield in alkaline medium is always less than 25%, however, the %H_2_O_2_ yield is between 10 and 35% in 0.5 M H_2_SO_4_. The Tafel plots at pH 0.3, 5.2, and 13.0 electrolytes are shown in Figure 4a–c, respectively. In a pH = 0.3 electrolyte, both iron and cobalt catalysts perform much better than others, which form a tight cluster of performance except for bismuth which shows a poorer performance among all (although with a similar shape). Although both Fe and to a lesser extent Co do not develop a well-defined Tafel slope, we display a line with a slope of 45 mV decade^–1^ so the results can be compared to the alkaline case. For the other catalysts that cluster together, a similar performance is seen with a high value of the Tafel slope (a line of 150 mV decade^–1^ is shown for comparison). For the acetate buffer electrolyte (pH = 5.2), a similar trend was observed as the acid case. All catalysts had very similar shapes and performances, except iron, cobalt, and bismuth. Cobalt showed a Tafel slope at around 45 mV decade^–1^ for around two orders of magnitude and iron has a slightly higher Tafel slope without a well-defined linear portion. The similar Tafel slope for cobalt and iron over the electrolytes with different pH values suggested the same number of electrons transferred in the rate-determining step (RDS). However, for other catalysts clustering together, they all showed varied Tafel slopes under different electrolytes, indicating the change of the rate-determining step (RDS) with pH. The best performance in 0.1 M KOH was seen for Fe with the other catalysts clustering together in a band. Close examination of the results in KOH shows that Fe and Co demonstrate a Tafel slope of close to 45 mV decade^–1^ at high potentials and in a range spanning more than two orders of magnitude of current density (NB takes about two orders of magnitude in current for a valid Tafel slope to appear). Other catalysts show a continuous variation in slope with no clear Tafel region, suggesting that there is a change in RDS over that range of potentials. For illustration, a line with a Tafel slope of 60 mV decade^–1^ is marked showing that all other catalysts require relatively little overpotential to accelerate the ORR in the high potential region (*E* > 0.7 V_RHE_). Because of the high performance of the catalysts, mass transport to and within the catalyst layer complicates the analysis as correction to kinetic current encounters large errors once the current is a significant fraction of the mass transport limiting current (5 mA cm^–2^ in these experiments).

The comparison of mass activity at 0.80 V_RHE_, which is calculated from kinetic current, is shown in Figure 4d. Clearly, the mass activity in an acidic medium is much poorer than that in an alkaline medium at the same voltage (∼1000-fold lower at the same potential). In acidic conditions, the mass activities of most catalysts at 0.80 V_RHE_ were similar to that of the (Zn)–N/C catalyst except iron, cobalt, and bismuth-based catalysts. Those of Fe–N/C, Co–N/C, and Bi–N/C were 30-fold higher, 5-fold higher, and 10 times lower respectively, compared with the mass activity of (Zn)–N/C. As the activity does not appear to significantly change for catalysts composed of Zn, Ni, Mn, Sn, and Sb, it is reasonable to assume that those metals do not appreciably affect the performance of the catalyst. Hence, the average mass activity (∼0.1 A g^–1^ in pH = 0.3 shown as a pale gray dashed line) should be the framework mass activity of this kind of catalyst determined by the N/C framework. Although the average mass activity in acetate buffer solution (∼0.08 A g^–1^) is slightly smaller than the mass activity in sulfuric acid, they had a similar trend, in which those of iron and cobalt were 25-fold and 5-fold higher, respectively, compared with the framework activity of the N/C framework. In alkaline conditions, only Fe–N/C and Co–N/C catalysts showed higher (6-fold higher performance) than the average values estimated from the (Zn)–N/C material, whereas that of bismuth was 1/3 of the performance of that material. In alkaline conditions, the major ORR activity appears to be controlled by the N/C framework as all catalysts had quite a high mass activity (∼50 Ag^–1^) at 0.80 V_RHE_.

Metal ions play a more important role at lower pH, including sulfuric acid and acetate buffer solution, because the difference in mass activities between iron or cobalt and other catalysts is greater than that in alkaline conditions. The mass activities in alkaline conditions are much higher than in acidic conditions, which suggests that the mechanism of ORR depends on the pH of the electrolyte. In alkaline conditions, some metal-free N/C catalysts reported in the literature also showed a very good activity and they all suggested that the amount of pyridinic N potentially influences the alkaline ORR activities.^58,59^ For our materials, around 50 atom % of nitrogen is pyridinic nitrogen as shown in the XPS (Supporting Table S3); so this should be the reason why all our catalysts showed relatively high ORR performance in the alkaline medium. The p*K*_a_ of pyridinic nitrogen is around 5.2, which suggested that the nitrogen sites could be protonated under acidic conditions, and hence, show a lower ORR activity.^60^ Moreover, according to the kinetic isotope effect (KIE) study performed on similar types of catalysts, an outer-sphere electron transfer process is more likely to take place in an alkaline electrolyte, which does not require direct adsorption of oxygen molecules on the surface of the catalyst.^61,62^ This also suggests that metal centers are not as crucial for alkaline ORR as they are for acidic ORR where oxygen needs to be adsorbed on the metal centers (an inner-sphere mechanism).

### Nitrite Stripping to Quantify Active Sites in Different M–N/C Catalysts

**Figure 5:**
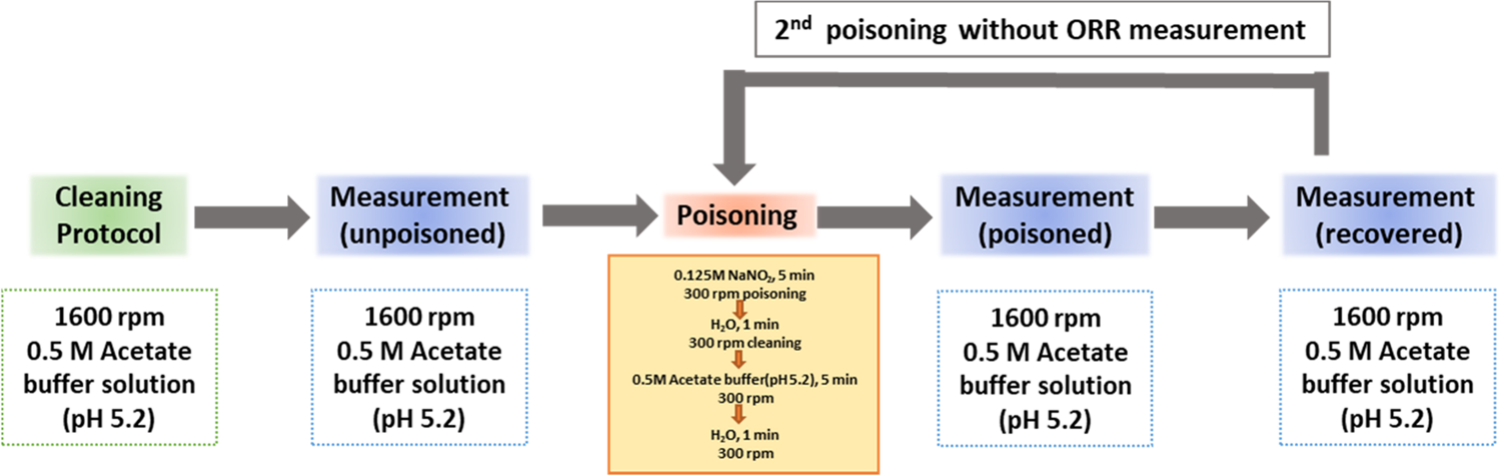
Protocol of the nitrite stripping technique to estimate the active site density: white box stands for electrochemical measurement and the orange box is a chemical process.

**Figure 6:**
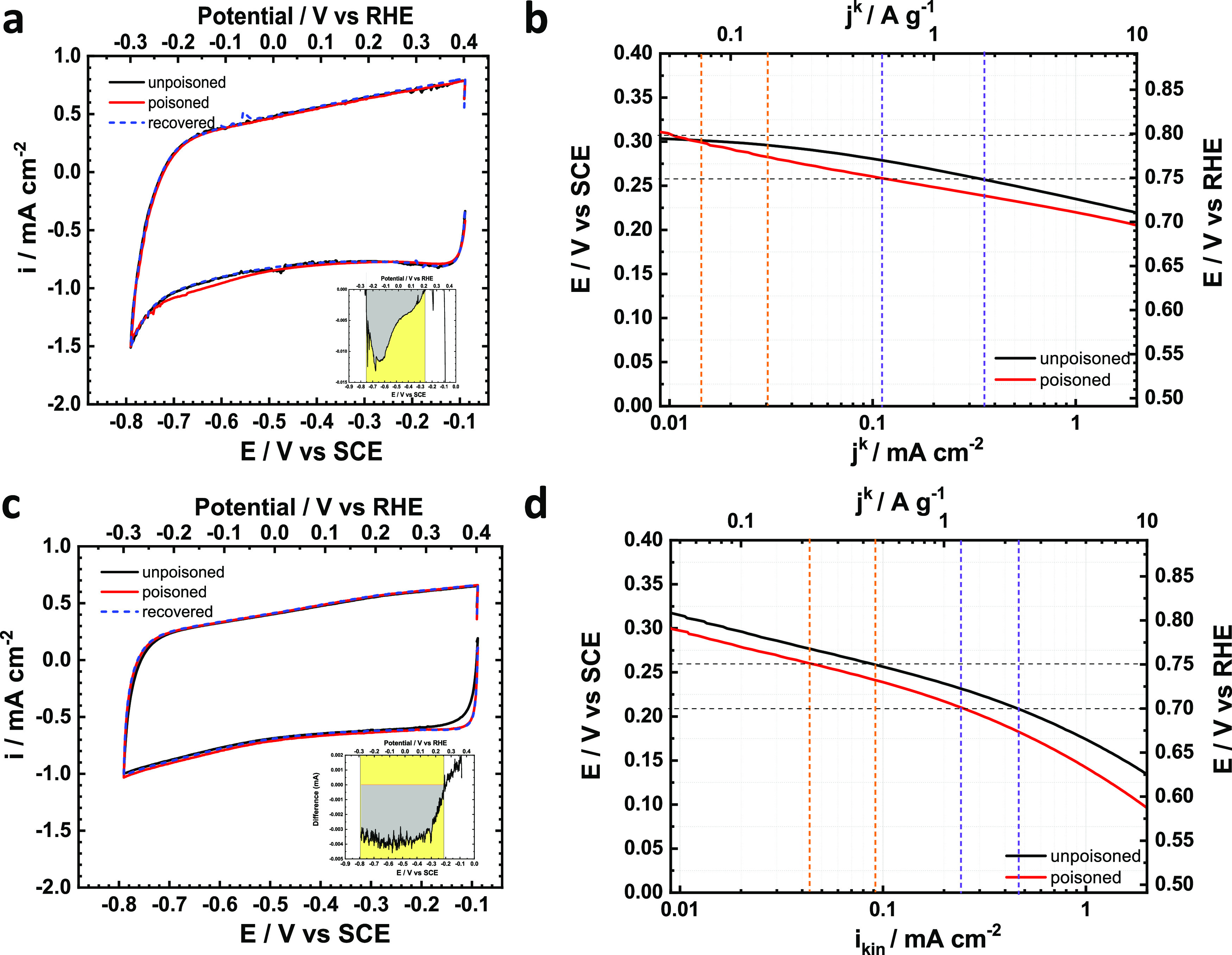
Nitrite stripping plots of M–N/Cs. (a, c) Baseline CV scans in nitrite reductive stripping with unpoisoned (black), nitrite poisoned (red), and recovered (blue dash) catalyst. Inset shows the reductive stripping curve by subtracting the recovered from poisoned curve; (b, d) negative shift of kinetic ORR activities upon nitrite poisoning unpoisoned (black), nitrite poisoned (red), and (a, b) Co–N/C and (c, d) Sb–N/C.

The measurement protocol of nitrite stripping is shown in Figure 5. Some small modifications have been introduced to measure the site density more accurately and the detailed procedure is provided in Supporting Figure S1.^16^ A current integrator was used for nitrogen CV to determine the stripping charge more accurately. Nitrite stripping is used to determine the active site densities in seven different M–N/Cs including Fe, Co, Ni, Mn, Sn, Sb, and Bi. Two representative examples of Co–N/C and Sb–N/C are shown in Figure 6 and detailed ORR curves and nitrogen CVs before and after nitrite poisoning are shown in Supporting Figures S18–S24. A nitrite reduction peak can be observed at around −0.2 V_RHE_. in the baseline CVs shown in Figure 6a,6c. The site density values are calculated based on eq 2 using the stripping charge and the change in kinetic current at a given potential, for instance at 0.75 or 0.80 V_RHE_, is used to determine the turnover frequency (TOF) according to eq 3. The summarized site density and TOF_total_ values at 0.75 V_RHE_ are shown in Table 1.

The site density values listed in Table 1 reveal that most of catalysts except iron and cobalt interestingly have a very similar number of active sites. This observation fits well with their very similar ORR performances in acidic and alkaline electrolytes as discussed earlier. As the ORR activities and site densities of these M–N/C catalysts with the exception of Fe–N/C and Co–N/C match closely to the reference (Zn)–N/C framework despite vastly different loadings of coordinated metal centers (e.g., 0.18 wt % Mn in Mn–N/C vs 3.14 wt % Sb in Sb–N/C), this suggests that nitrite adsorption and reduction is possibly occurring at non-metal N/C framework sites in those catalysts. A comparative plot of NO stripping currents for all M–N/Cs is shown in Supporting Figure S25 where only Fe–N/C and Co–N/C show a distinct reduction peak at around 0.05 and −0.15 V_RHE_, respectively. The absence of any well-defined nitrite reductive peaks for the rest of M–N/C catalysts supports our assumption that nitrite is likely to be adsorbed (and reduced) on the N/C framework without any detectable contribution of metal sites in those catalysts. The number of N/C framework sites (framework site density) is quantified by averaging the stripping charge of all catalysts except Fe–N/C and Co–N/C. The average value of stripping charge for all other catalysts is 3.9 ± 0.9 C g^–1^, which corresponds to a framework site density of 4.9 × 10^18^ sites g^–1^. This calculated value of framework site density for the N/C framework is very close to the number of sites (SD_0_ = 6.2 × 10^18^ sites g^–1^) in Fe–N/C that we reported in our recent study as ORR inactive in the high potential region (>0.80 V_RHE_) in an acidic electrolyte (see Figure 5b and related discussion in that paper).^16^ Furthermore, there is still around 40% ORR kinetic current retained after poisoning (see Figure 6d as an example) indicating that there are additional framework N/C sites (around 40%) that are not poisoned by nitrite. If those unpoisoned N/C sites are assumed to be the exactly same as the poisoned ones, then the framework site density value becomes 7.8 × 10^18^ sites g^–1^.

**Figure 7:**
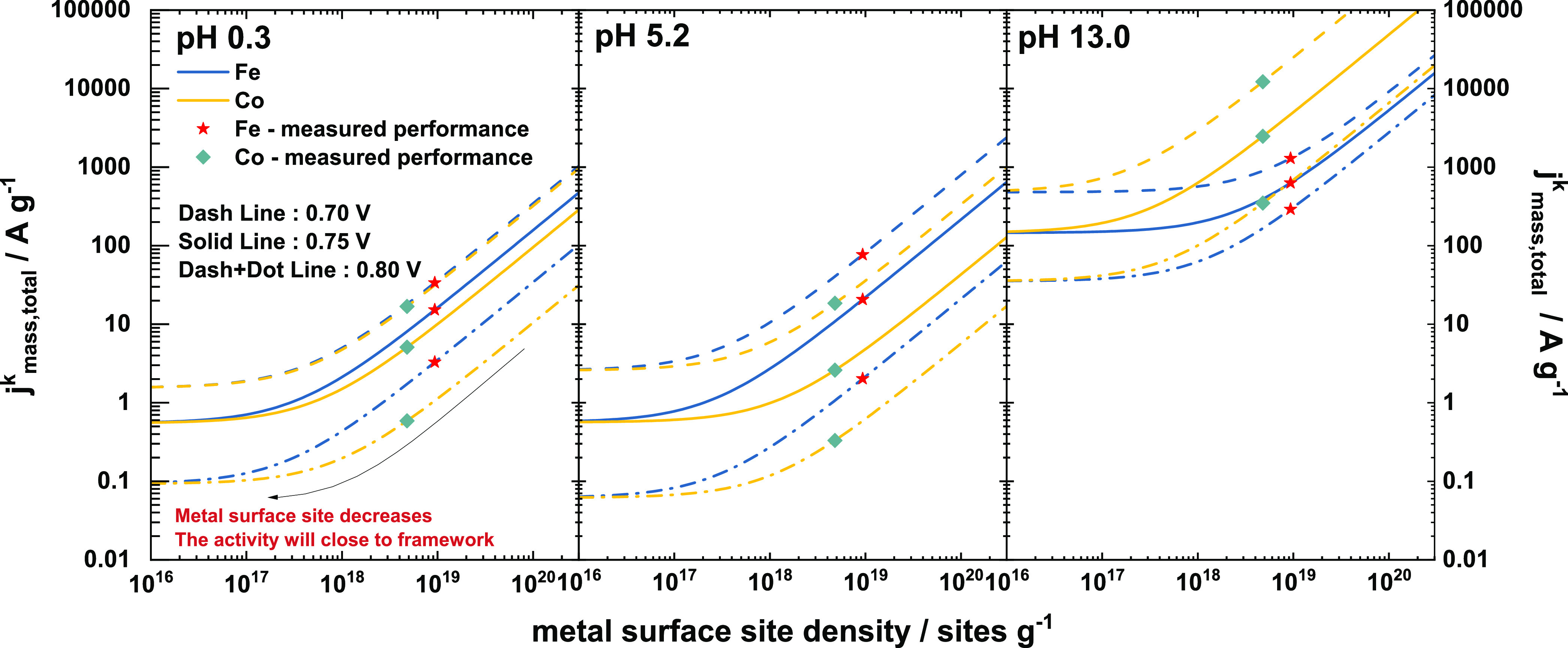
Relationship between the metal surface density and the kinetic mass activity in three different pH electrolytes at 0.70, 0.75, and 0.80 V_RHE_: Stars are the measured Fe–N/C performance in this work and rhombuses stands for the measured Co–N/C result. Lines, including dashed line, solid line, and dashed+dotted line, are the predicted *j*_mass,total_^k^ calculated from the metal surface site density.

As discussed above in Figure 4, both Fe–N/C and Co–N/C show considerably higher ORR activity in different pH electrolytes as compared to the other M–N/C catalysts. Moreover, the site densities of these two catalysts are around 2–3 times higher than the N/C framework site density. This allows us to deconvolute the activity of catalysts in terms of framework sites’ and metal sites’ contribution. The kinetic mass activity (i.e., in the absence of mass transport effects) of the catalyst at a given potential, pH, and containing a given coordinated metal is composed of two components, one associated with the activity of the metal sites, and one associated with the activity of the framework. The framework activity is common across all the catalysts whereas we have shown that the mass activity of the metal sites is only non-zero for Fe and Co catalysts. Therefore, the kinetic mass activity could be calculated by eq 6.
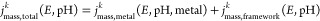
6We can break down each of the mass activity components into their respective site densities (SDs) and turnover frequencies (TOFs) as shown in eqs 7 and 8.
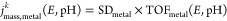
7
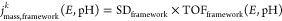
8Hence, the measured mass activity is dependent on four independent parameters—two site densities and two turnover frequencies. However, the question now comes as to whether we can independently estimate the values of each of these different parameters. It is assumed that site density for both metal and framework sites does not change with potential or pH in eqs 7 and 8 i.e., we attribute all the changes in mass activity of the sites when changing potential and pH as due to a change in TOF. *j*_mass,framework_^*k*^ could be estimated by the average performance of all catalysts apart from the Fe–N/C and Co–N/C ones, as they all show the same activity. Thus, it appears that the metal has no effect on the performance, suggesting that all the activity comes from the N/C framework. For Fe–N/C and Co–N/C, the extra mass activity noticed must be due to the metal sites, therefore *j*_mass,metal_^*k*^ for Fe and Co catalysts could be calculated by the difference between *j*_mass,total_^*k*^ and *j*_mass,framework_^*k*^. As the SD_metal_ values were estimated from nitrite stripping, this also allows us to determine the TOF_metal_. The calculated SD_framework_, SD_metal_, TOF_framework_, and TOF_metal_ values as listed in Supporting Table S6. Both Fe and Co see significantly more nitrite adsorbed than the other samples, but interestingly we do see some nitrite absorption associated with all samples, corresponding to on average 4.9 × 10^18^ sites g^–1^. We thus need to correct the measured metal site density for these “extra” absorption sites. Some framework N/C sites are poisoned by nitrite and some are not, which suggests that eq 6 may need to be further modified to describe two different types of framework sites. Figure 7 displays these values using the determined mass activity of the framework (the asymptotic minimum value seen as metal site density decreases), and the measured catalyst mass activity associated with the determined active metal site density (stars for Fe–N/C and rhombuses for Co–N/C). The lines show the expected form of the curves as metal site density is changed.

A comparison of TOF of different sites in different electrolytes (Supporting Table S6) clearly shows that the framework sites are considerably less active under acidic conditions as TOF_framework_ values are much smaller than those of the Fe–N/C and Co–N/C sites. Iron sites (Fe–N_4_) are the most active in pH 0.3 and pH 5.2 electrolytes with their TOF values 2–4 times higher than those of the cobalt sites (Co–N_4_). However, cobalt sites (Co–N_4_) give substantially higher activity under alkaline conditions outperforming Fe–N_4_ sites at all potentials. The framework N/C sites are also significantly more active in an alkaline electrolyte as compared to their corresponding pH 0.3 and pH 5.2 TOF_framework_ values. Based on the TOF_framework_ and TOF_metal_ values calculated under different pH conditions, the ORR kinetic mass activity can be predicted if the metal surface site density is known as shown in Figure 7. The stars represent measured performance data points for Fe–N/C and rhombuses stand for the measured activity of Co–N/C in this work. If the metal surface site density in the M–N/C catalyst is very small, then the catalyst will only show the framework activity, however, increasing the metal surface site density would lead to a significantly higher ORR mass activity.

## Conclusions

We successfully prepared several different M–N/C single-atom catalysts with M = Fe, Co, Ni, Mn, Sn, Sb, and Bi by our recently reported ZIF-8-based (trans)metalation approach. The benefit of this synthetic route is that all catalysts have a nearly identical N/C framework structure and based on XAS analysis, all prepared M–N/C catalysts consisted of M–N_4_ sites with no other detectable side phases. This allowed us to compare the framework ORR activity of different active metal centers in different pH electrolytes in a relatively fair manner with minimal interference of morphological effects of the carbon framework. ORR activity in different pH electrolytes has been tested for all catalysts, including pH 0.3, 5.2, and 13.0. All catalysts exhibited higher ORR activity in alkaline conditions than in acidic and near-neutral electrolytes. In an acidic medium, the iron and cobalt metal sites (Fe–N_4_ and Co–N_4_) showed significantly high ORR activity, while rest of the tested single-atom metal sites were inactive toward ORR. Interestingly, despite a substantial variation in the loading amounts of Ni, Mn, Sn, Sb, and Bi in the N/C framework, the ORR activity did not show a noticeable variation, suggesting the existence of non-metallic N/C framework sites. The ORR activity of these framework sites in the acidic electrolyte was poor particularly in the high potential region (>0.75 V_RHE_) and significantly lower than those of the Fe–N_4_ and Co–N_4_ sites. However, in alkaline conditions the ORR activity of framework sites improved greatly, although still lower than the Fe and Co-based active sites. The existence of the N/C framework sites was further supported by active site density measurements using nitrite stripping, where all M–N/C catalysts except for Fe–N/ and Co–N/C showed similar site density values despite significantly different metal loadings. Based on nitrite stripping results, an average framework site density value of 4.89 × 10^18^ sites g^–1^ was calculated. The calculated value of site density for N/C framework sites matched closely to the number of ORR sites in Fe–N/C that we recently reported as inactive in the high potential region in an acidic medium. Based on the new results presented in this work, it is now clear that those sites are indeed the non-metallic framework sites, which were up until now difficult to discern from M–N_4_ sites due to the limitations of site density quantification methods as well as difficulties in preparing M–N/C catalysts with well-defined active sites and equivalent morphological structures.

## Data Availability

The data used in the production of the figures in this paper are available for download at DOI: 10.5281/zenodo.7879880.

## Supporting Information Available

Protocols for electrochemical testing. For all the catalysts tested: TEM and elemental mapping; SEM and EDS analysis; XANES and EXAFS results and analysis; XPS, Raman and BET; ORR and background voltammetry; nitrite stripping and kinetic ORR analysis; TOF and site density analysis (PDF)
