# Supplementary material for: Oxygen Reduction Reaction Activity in Non-Precious Single-Atom (M–N/C) Catalysts—Contribution of Metal and Carbon/Nitrogen Framework-Based Sites
Source: ACS Catal. 2023 May 1;13(10):6661–74. doi: 10.1021/acscatal.3c00356 (PMC10204730; doi:10.1021/acscatal.3c00356)
Supplement: Supplementary file 1 — cs3c00356_si_001.pdf [file cs3c00356_si_001.pdf]

Supplementary Information

Oxygen reduction reaction activity in non-precious single atom (M-N/C) catalysts – contribution of metal and carbon/nitrogen framework-based sites

Mengjun Gong[a], Asad Mehmood[b], Basit Ali[c], Kyung-Wan Nam[c] and Anthony Kucernak[a*]

[a] Department of Chemistry, Imperial College London, White City Campus, London W12 0BZ, United Kingdom

[b] Division 3.6 – Electrochemical Energy Materials, Bundesanstalt für Materialforschung und -prüfung (BAM), 12203 Berlin, Germany

[c] Department of Energy and Materials Engineering, Dongguk University, Seoul 04620, Republic of Korea

*Correspondence to: Anthony Kucernak. Email: anthony@imperial.ac.uk

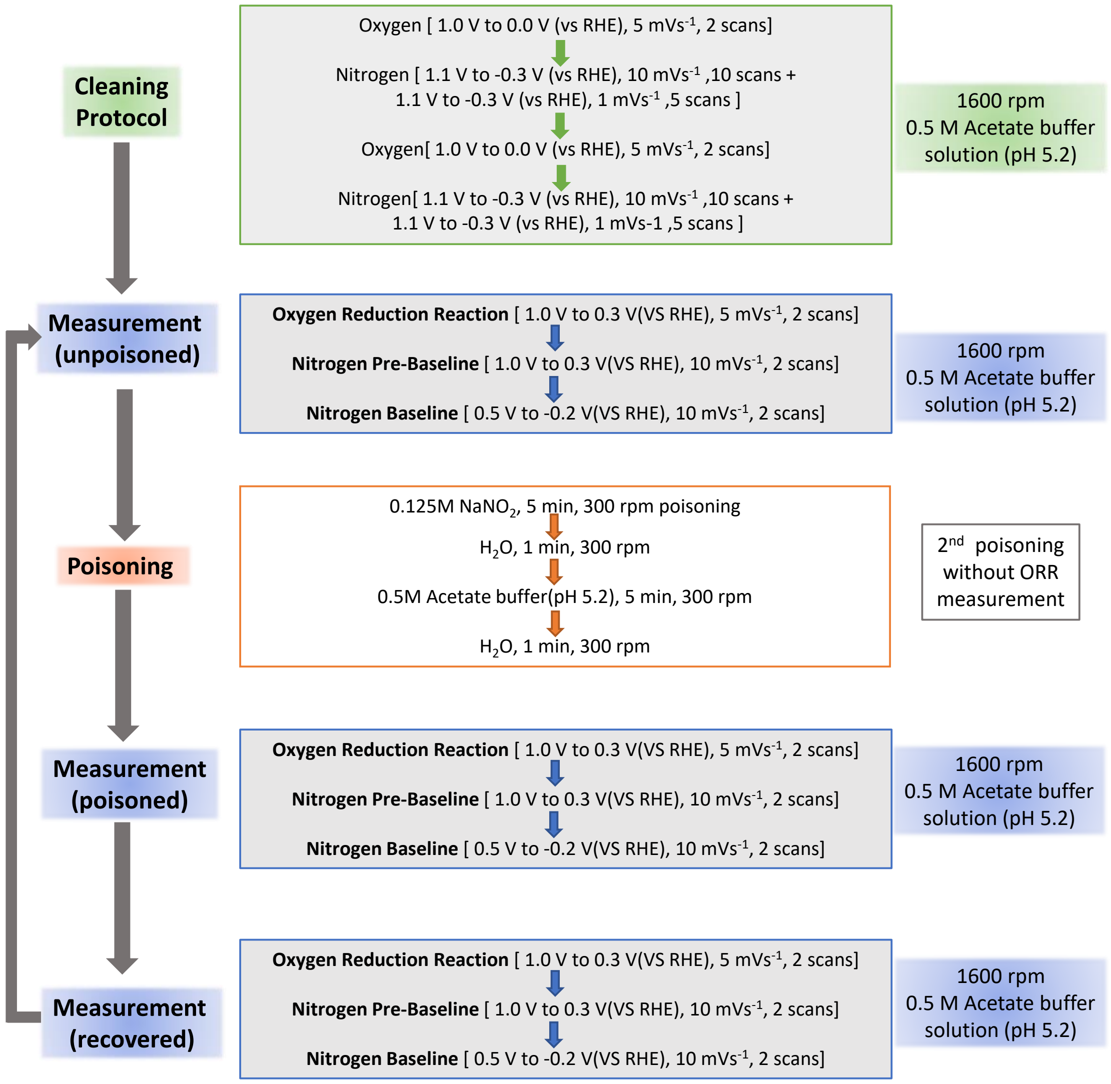

Fig S1. ¦ The schematic diagram of nitrite stripping method

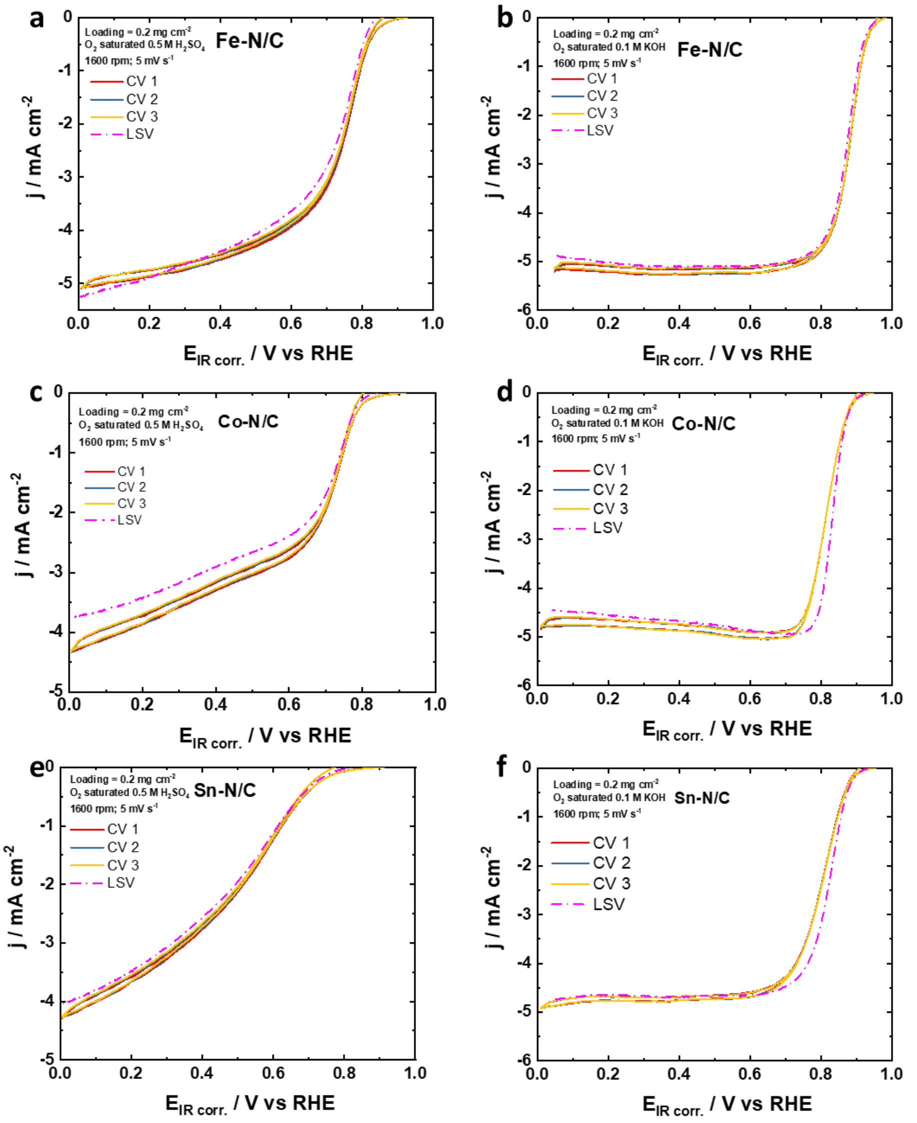

Fig S2. ¦ Comparison of activation CV and reported LSV of M-N/Cs in 0.5 M H_2SO_4 and 0.1 M KOH (a) Fe-N/C in 0.5 M H_2SO_4; (b) Fe-N/C in 0.1 M KOH; (c) Co-N/C in 0.5 M H_2SO_4; (d) Co-N/C in 0.1 M KOH; (e) Sn-N/C in 0.5 M H_2SO_4; (f) Sn-N/C in 0.1 M KOH.

For Pt group catalyst, a break-in is normally required to remove the potential oxide and active the catalyst. In this work, we also did performed three activation CV scans to make sure the catalyst is wetted properly, and the catalyst response is stable. In all cases, all CV activations overlapped each other very well, which indicates the catalyst is very stable and active. The CV's are not corrected using a N_2 saturated background. In contrast all the reported LSV are corrected by the LSVs performed under nitrogen. Therefore, there are slightly difference between CV and LSV.

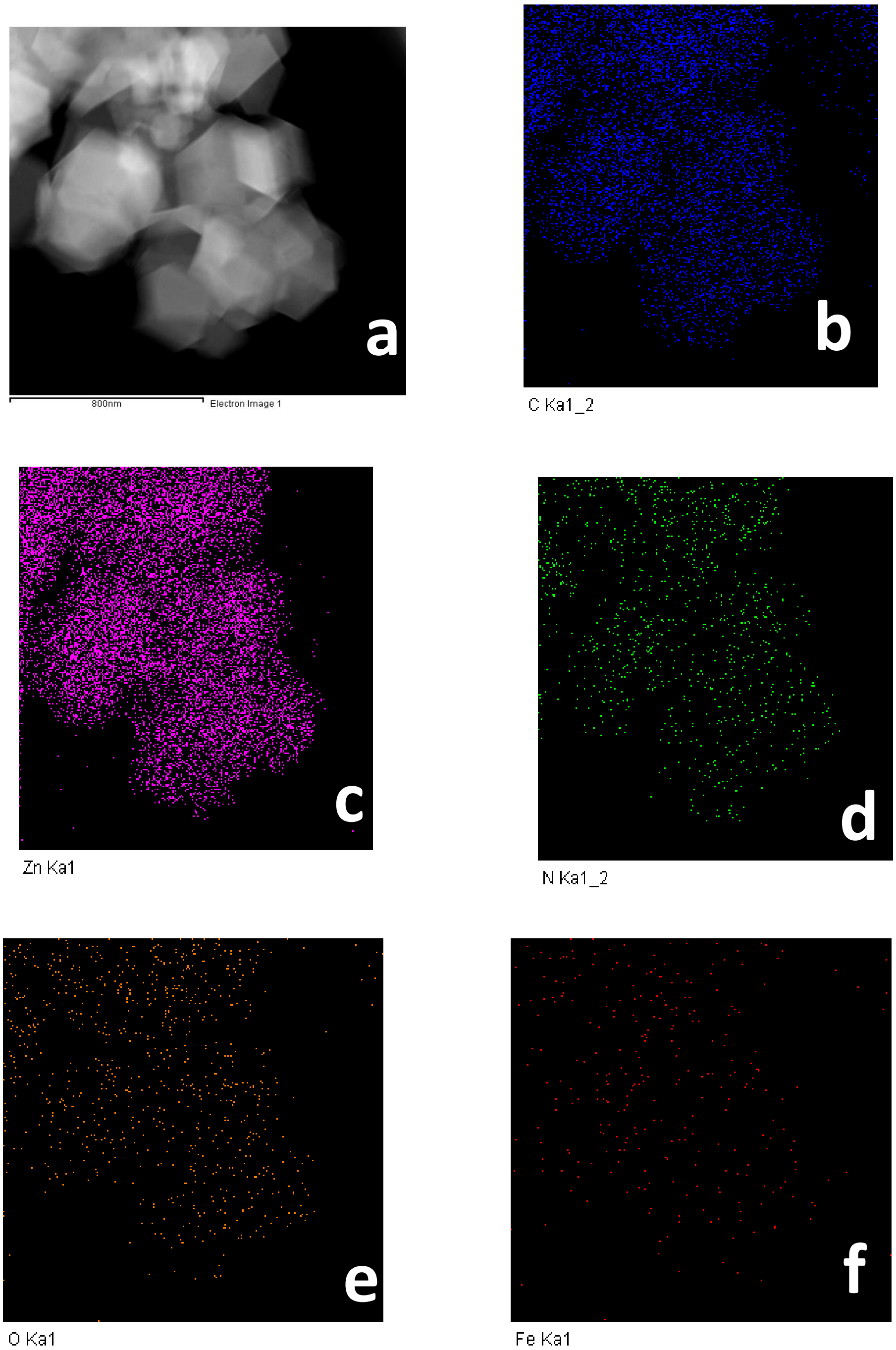

Fig S3. ¦ TEM and elemental mapping of Fe-N/C catalyst (a) TEM image (b) C mapping (c) Zn mapping (d) N mapping (e) O mapping (f) Fe mapping

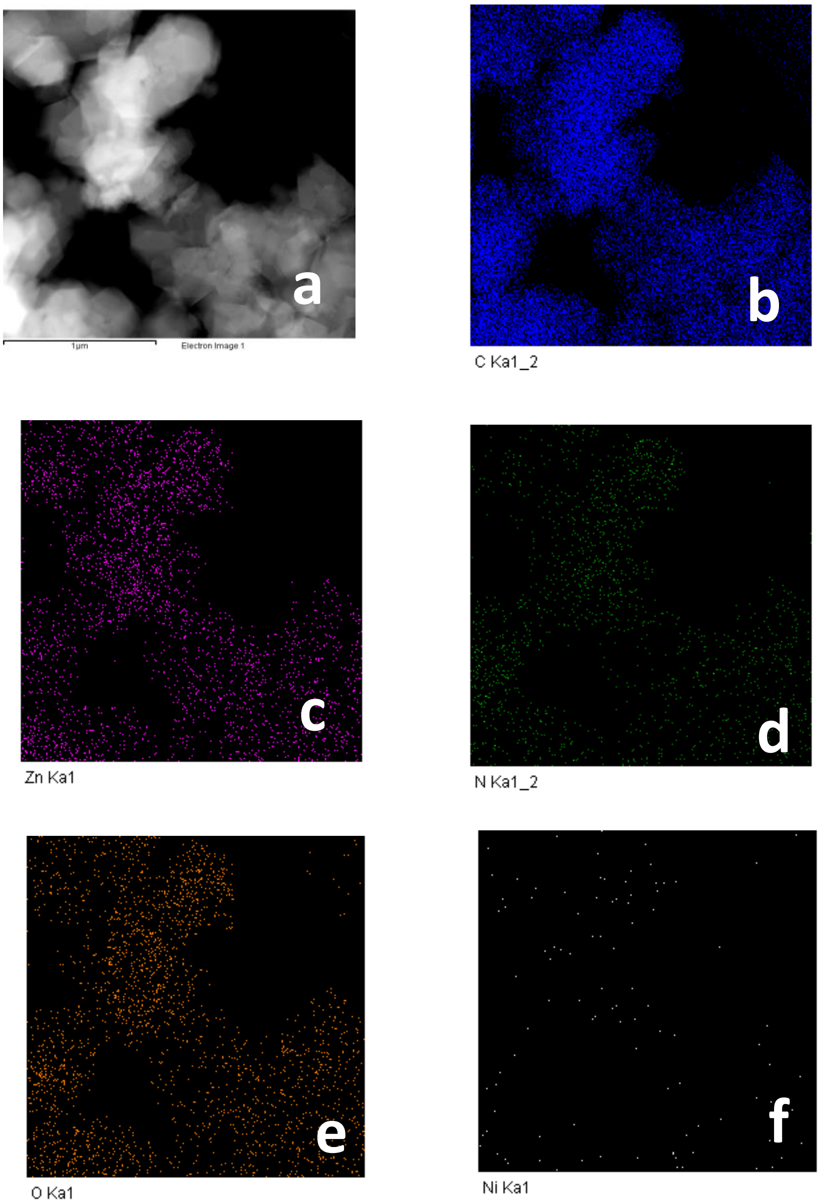

Fig S4. ¦ TEM and elemental mapping of Ni-N/C catalyst (a) TEM image (b) C mapping (c) Zn mapping (d) N mapping (e) O mapping (f) Ni mapping

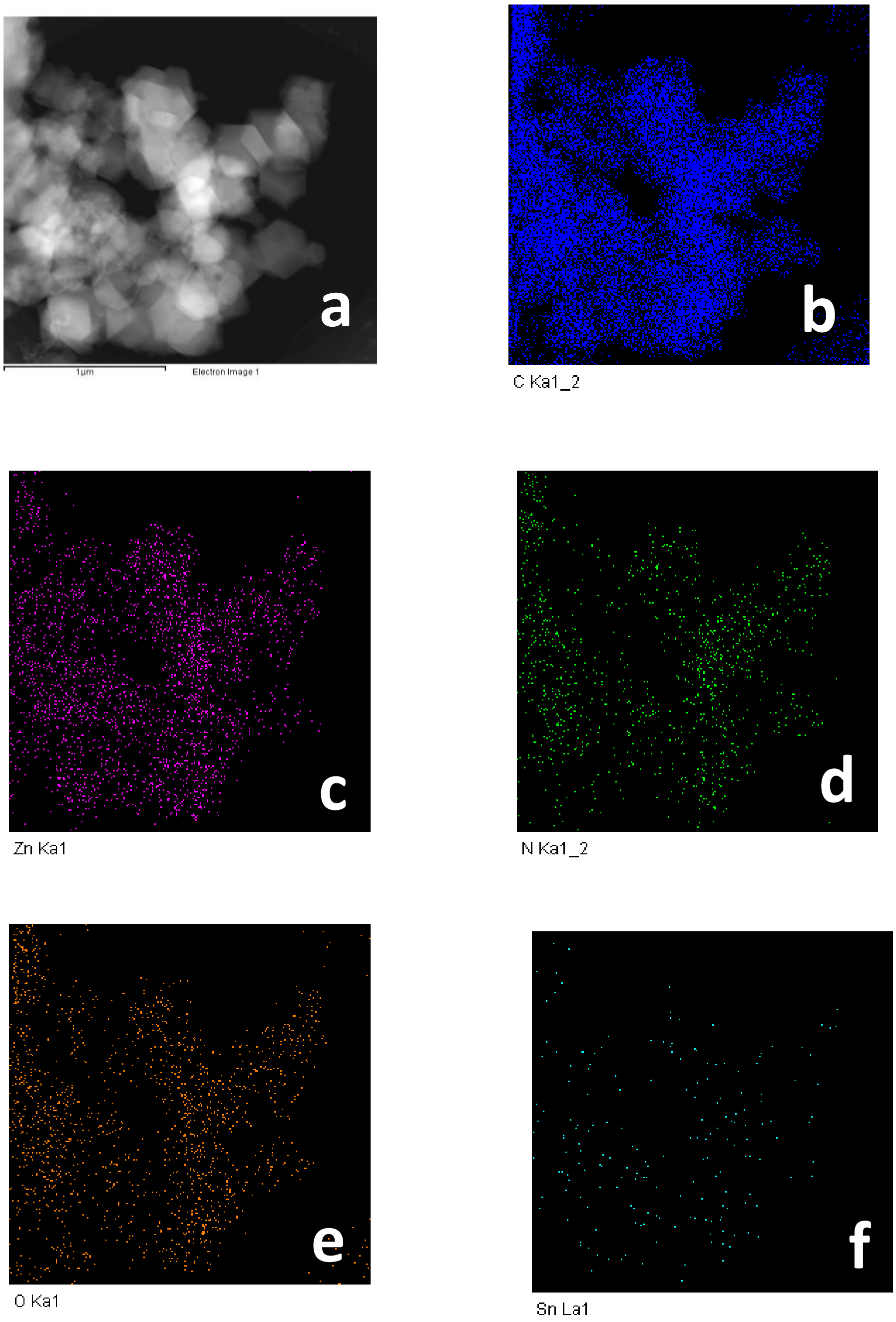

Fig S5. ¦ TEM and elemental mapping of Sn-N/C catalyst (a) TEM image (b) C mapping (c) Zn mapping (d) N mapping (e) O mapping (f) Sn mapping

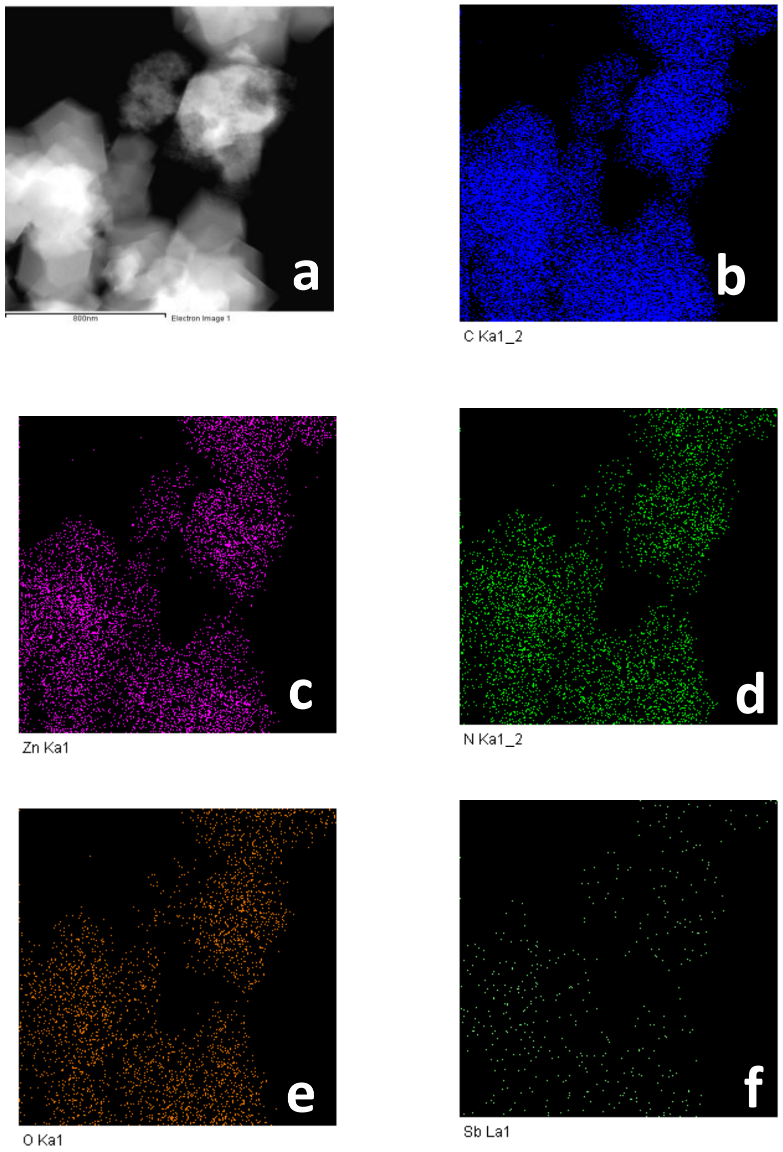

Fig S6. ¦ TEM and elemental mapping of Sb-N/C catalyst (a) TEM image (b) C mapping (c) Zn mapping (d) N mapping (e) O mapping (f) Sb mapping

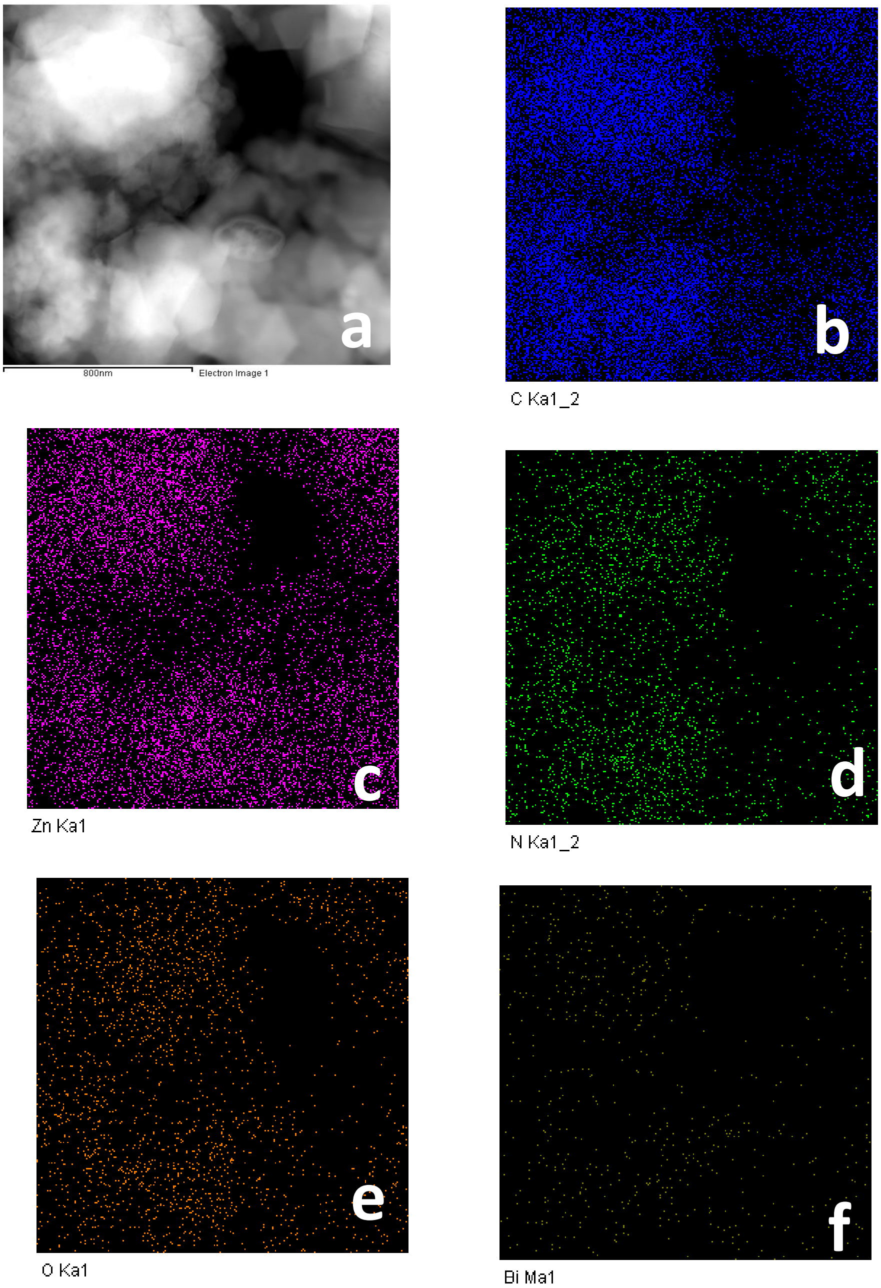

Fig S7. ¦ **TEM and elemental mapping of Bi-N/C catalyst** (a) TEM image (b) C mapping (c) Zn mapping (d) N mapping (e) O mapping (f) Bi mapping

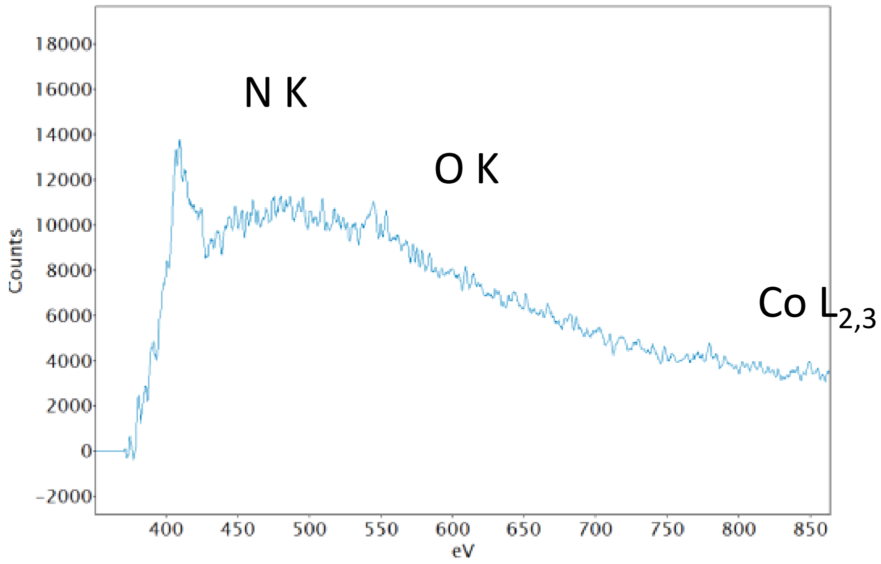

Fig S8. ¦ Electron energy loss spectroscopy (EELS) profile of Co-N/C catalyst.

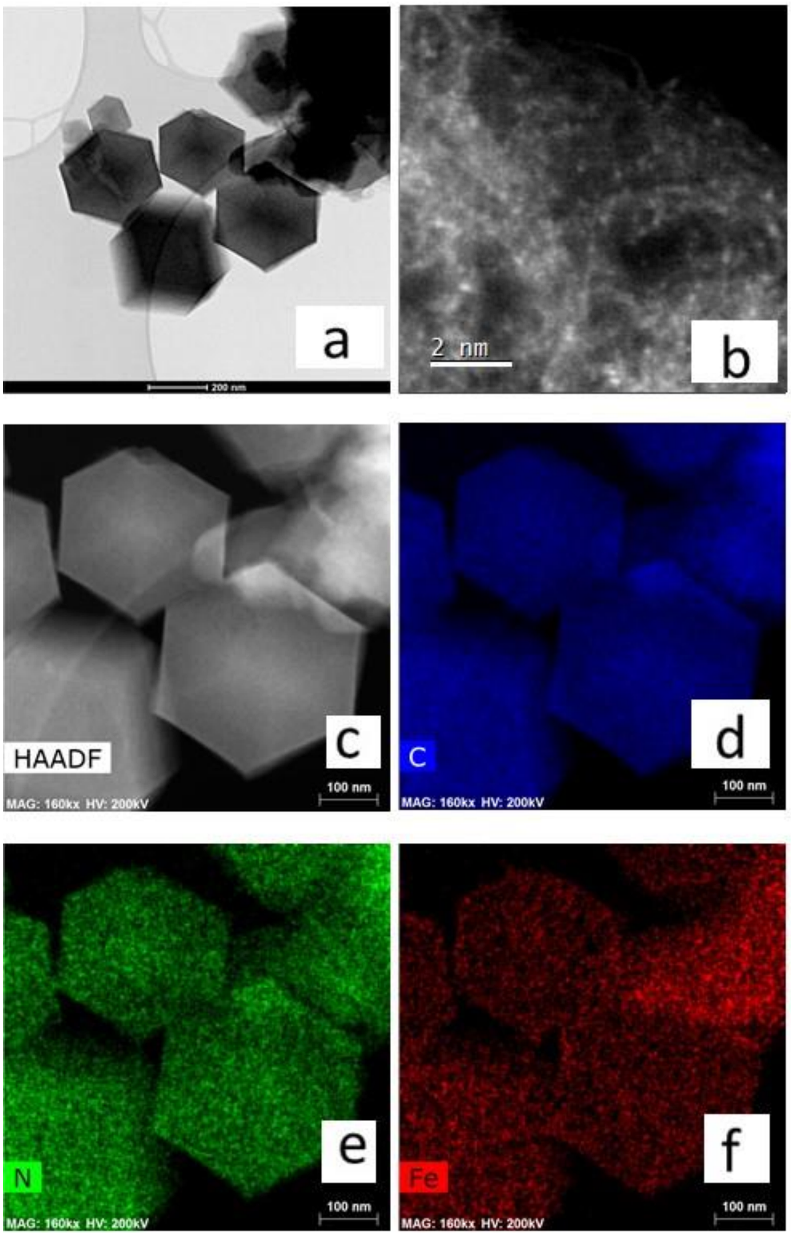

Fig S9. ¦ TEM and atomic resolution HAADF – STEM images and elemental mapping of Fe-N/C catalyst by EDS.[1]

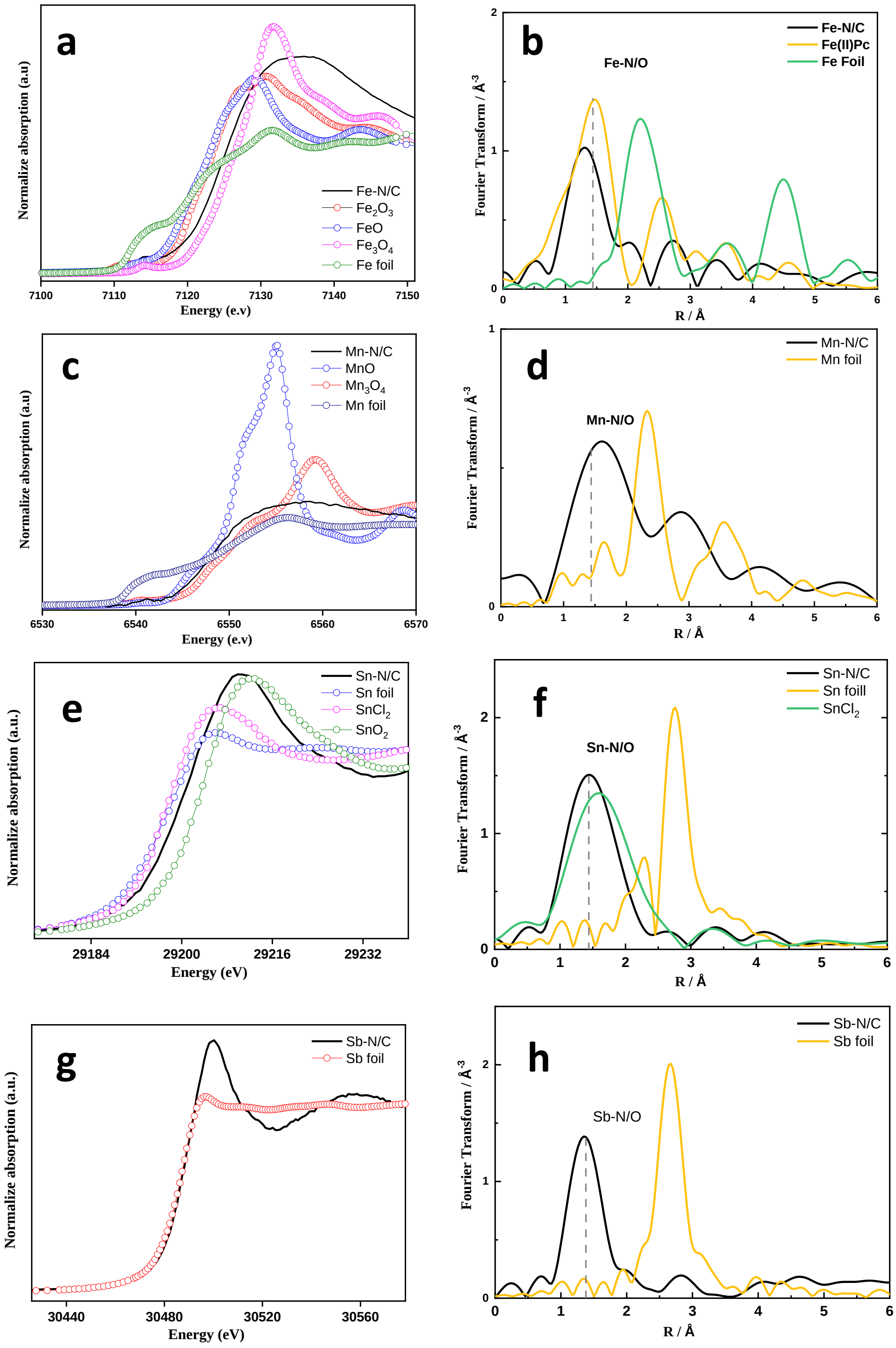

Fig S10. ¦ **XANES and EXAFS of M-N/Cs** (a) & (b) Fe-N/C; (c) & (d) Mn-N/C; (e) & (f) Sn-N/C and (g) & (h) Sb-N/C.

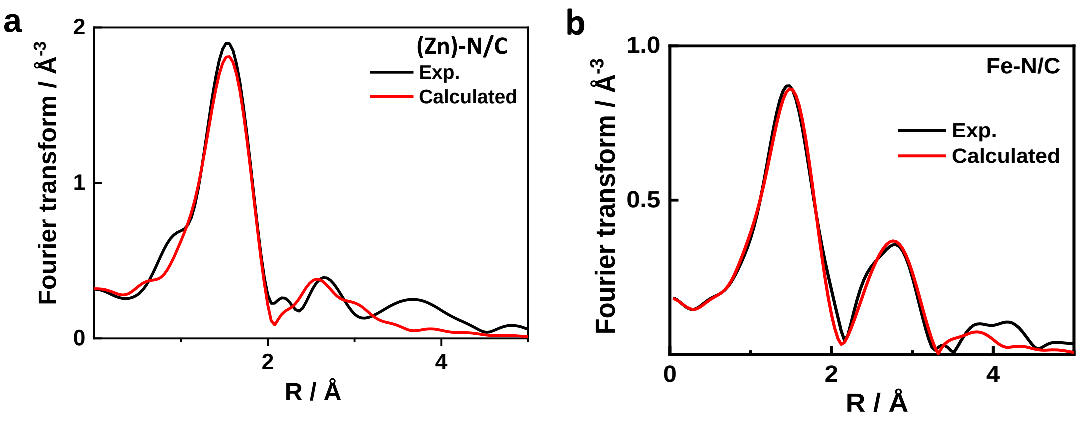

Fig S11. ¦ Zn / Fe K-edge EXAFS of Fe-N/C catalysts (a) (Zn)-N/C and (b) Fe-N/C in the Fourier transformed space without phase-shift correction. Black line represents the experimental data and red line is the calculated value.[1]

EXAFS fitting data is shown in Table S1 and the detail about fitting is shown in the Supporting Information of paper published by Mehmood *et al.*[1]

Table S1 ¦ Fitting of EXAFS of (Zn)-N/Cand Fe-N/C. Analysis based on the EXAF done in the work published by Mehmood *et al.* [1]

**Catalyst**		**Interatomic Distance / Å**	**Debye-Waller factor / 10^{-3} Å^2**	**Coordination number**
(Zn)-NC	Zn-N	2.02(1)	6.3(5)	4.0(2)
	Zn-C	3.19(2)	35(10)	6.2(1)
Fe-N/C	Fe-N	2.02(2)	9(2)	3.9(1)
	Fe-C_1	3.10(2)	12(2)	5(2)
	Fe-C_2	3.47(2)	11(2)	3(1)

For the further details about the EXAFS fitting, it is shown in Supporting Information in Nature Catalysis.

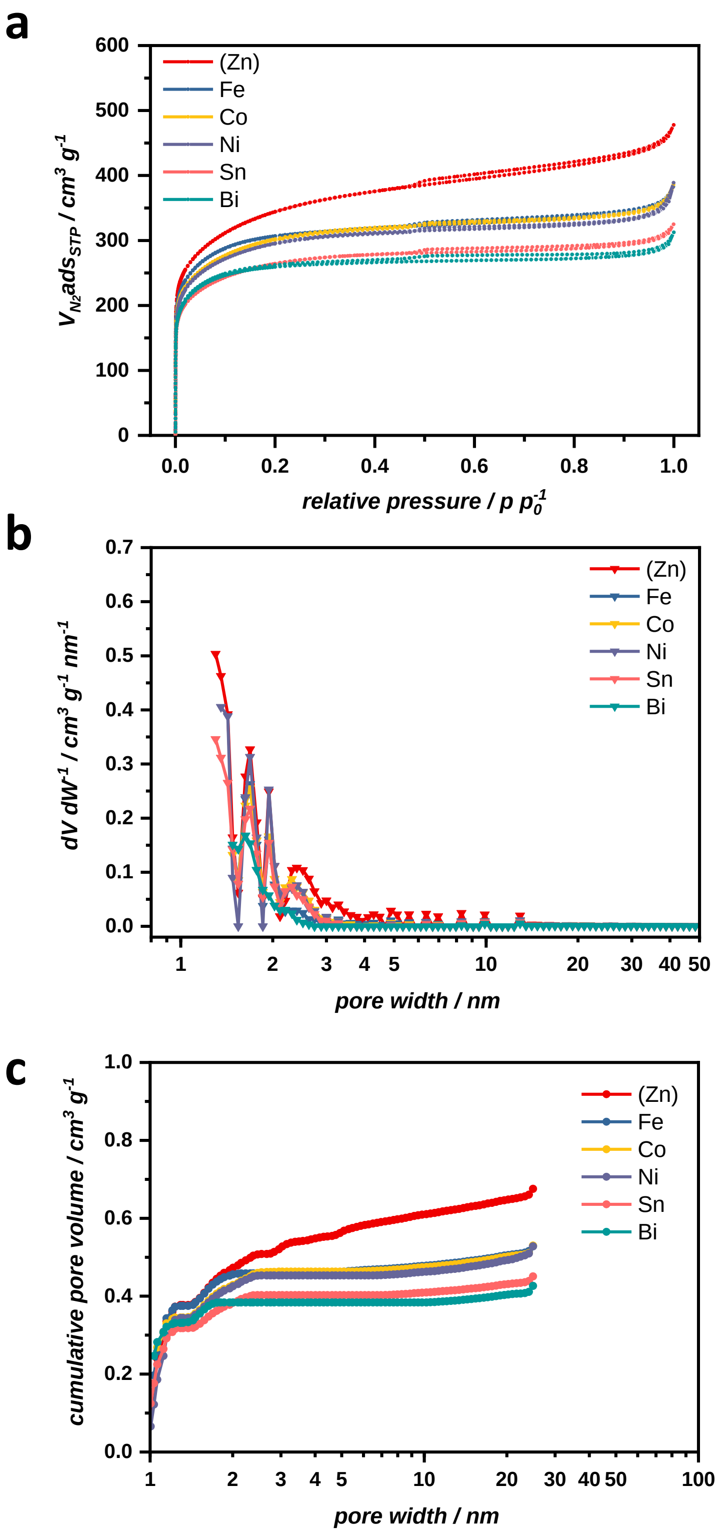

Fig S12. ¦ BET of M-N/Cs (a) N_2 adsorption and desorption isotherms; (b) pore size distribution profiles and (c) cumulative pore volumes.

Table S2 ¦ The BET surface area, microporous area and pore volume of M-N/Cs.

Catalyst	BET surface area / m^2g^{-1}	T-plot microporous Area/m^2g^{-1}	T-plot microporous Volume/cm^3g^{-1}	BJH Adsorption cumulative volume of pores /cm^3g^{-1}	BJH Desorption cumulative volume of pores/cm^3g^{-1}
(Zn)-N/C	1,239	877	0.374	0.363	0.370
Fe-N/C	1,145	997	0.408	0.191	0.196
Co-N/C	1,100	887	0.373	0.233	0.236
Ni-N/C	1,082	884	0.369	0.231	0.242
Sn-N/C	967	800	0.334	0.178	0.182
Bi-N/C	982	895	0.362	0.129	0.129

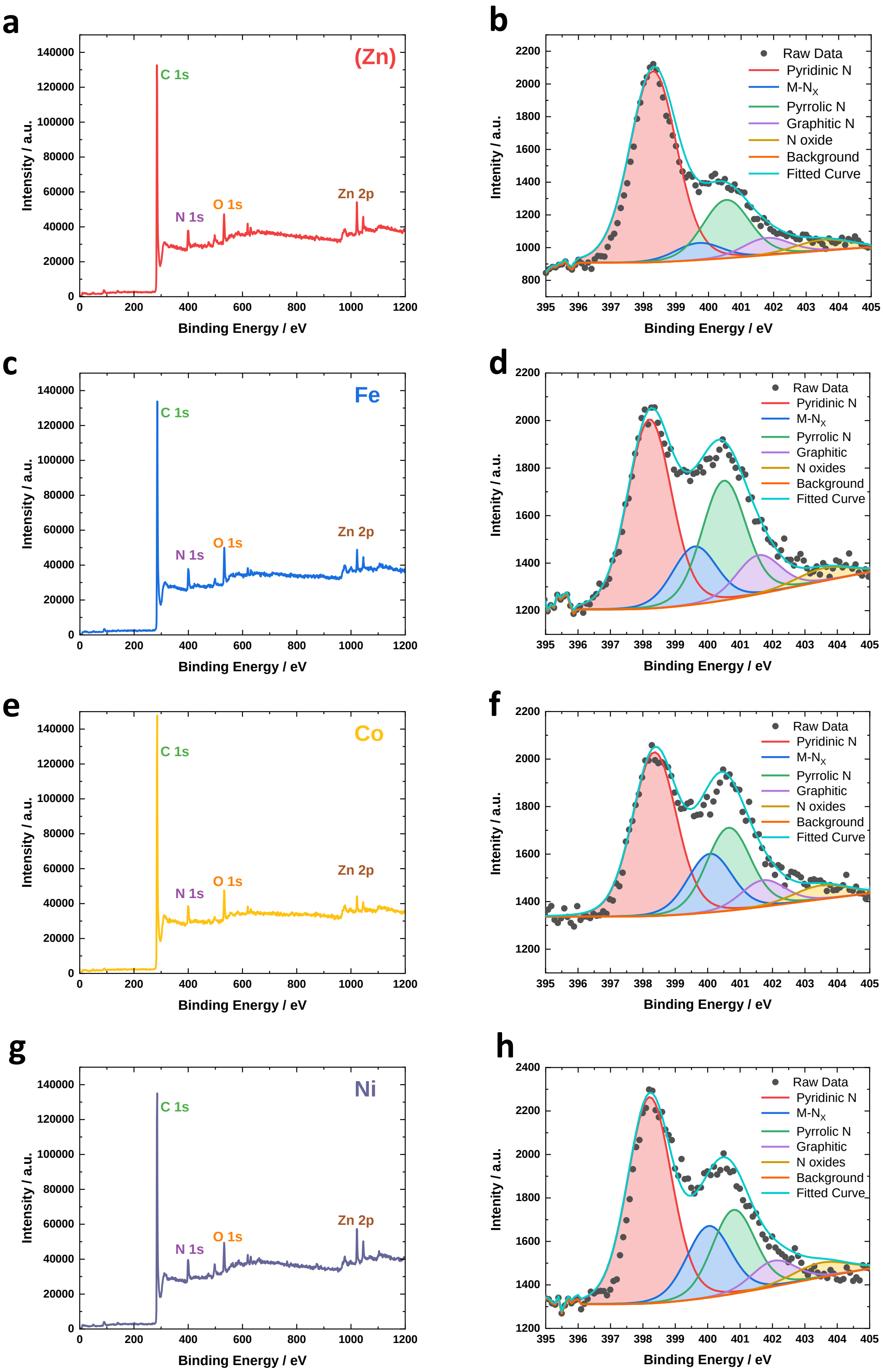

Fig S13. ¦ **XPS of M-N/Cs** (a), (c), (e) and (g) are the survey XPS spectra of (Zn)-N/C, Fe-N/C, Co-N/C and Ni-N/C catalysts respectively; (b), (d), (f) and (h) are the high-resolution N 1s XPS spectra of (Zn)-N/C, Fe-N/C, Co-N/C and Ni-N/C catalysts, respectively.

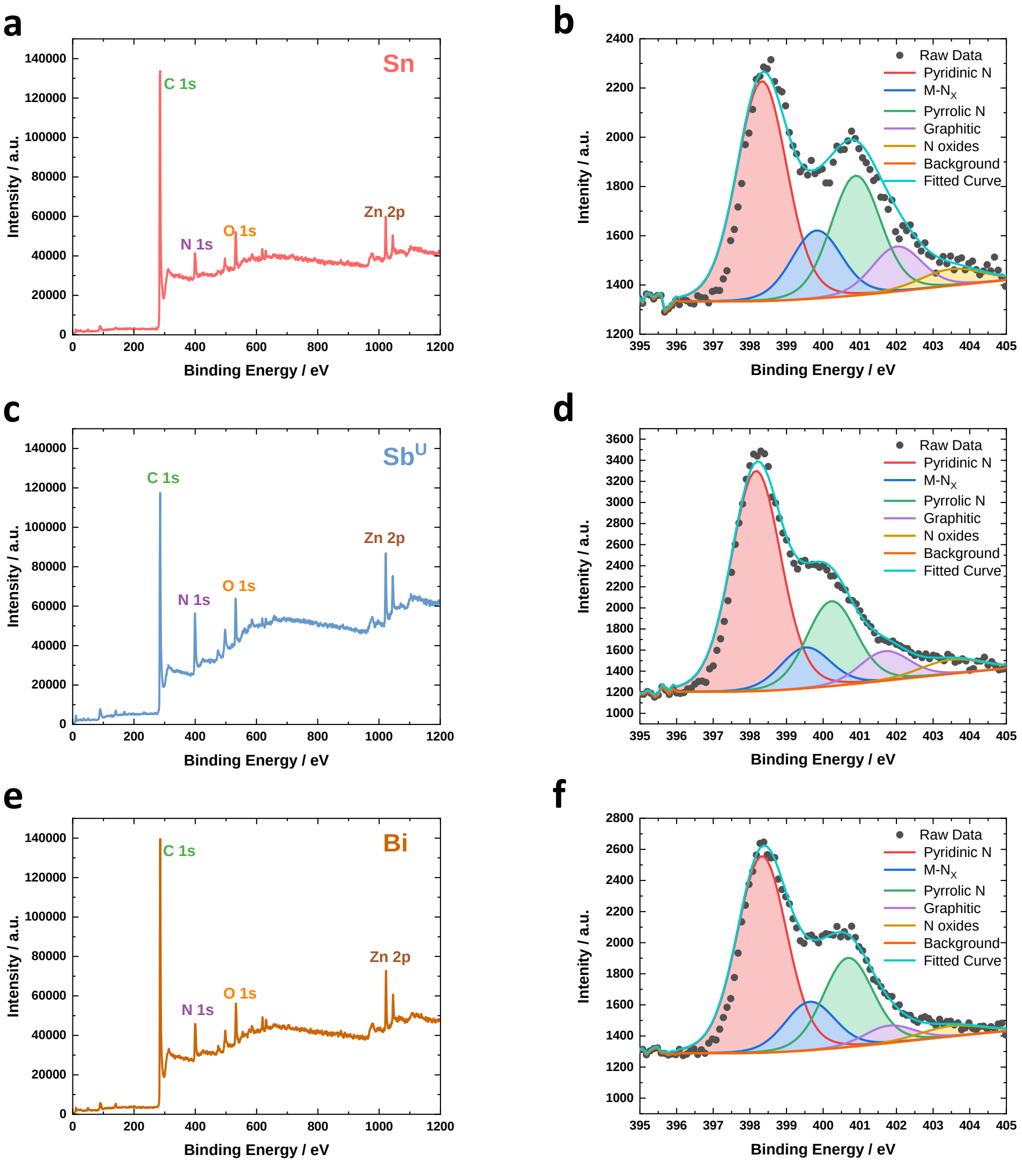

Fig S14. ¦ **XPS of M-N/Cs** (a), (c) and (e) are the survey XPS spectra of Sn-N/C, Sb^{U}-N/C and Bi-N/C catalysts respectively; (b), (d) and (f) are the high-resolution N 1s XPS spectra of Sn-N/C, Sb^{U}-N/C and Bi-N/C catalysts respectively. [^{U} stands for the unactivated catalyst, which means the catalyst was not pyrolysed under 5% of H_2/N_2 again.]

Table S3 ¦ The percentage amount of C, N, O, Zn and M in M-N/C. Analysis based on the XPS [^{U} stands for the unactivated catalyst, which means the catalyst was not pyrolysed under 5% of H_2/N_2 again.]

Catalyst/ at%	C	N	O	Zn	M
(Zn)-N/C^U	78.95	12.49	8.2	0.36	
(Zn)-N/C	89.69	6.25	3.71	0.35	
Fe-N/C	89.02	6.07	4.63	0.25	0.05
Co-N/C	91.23	4.47	4.05	0.17	0.09
Ni-N/C	89.46	6.18	3.88	0.47	
Sn-N/C	89.53	5.77	4.27	0.43	0
Sb-N/C^U	78.83	14.09	5.58	0.9	0.59
Bi-N/C	86.32	8.24	4.85	0.6	0

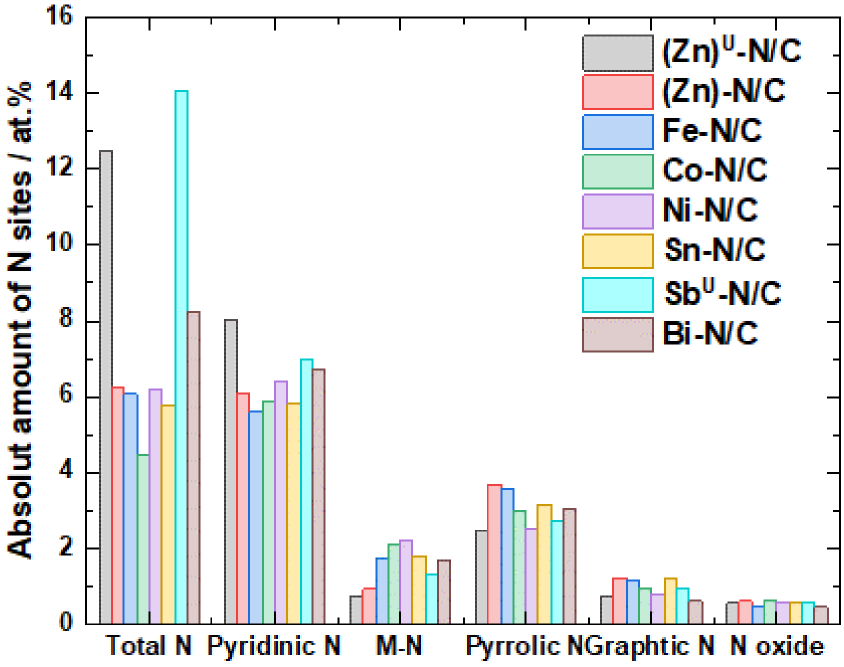

Fig S15. ¦ Absolute amounts of different nitrogen sites in the M-N/Cs. [U stands for the unactivated catalyst, which means the catalyst was not pyrolysed under 5% of H_2/N_2 again.]

Table S4 ¦ The percentages of different types of nitrogen in M-N/Cs. Based on the XPS analysis [^{U} stands for the unactivated catalyst, which means the catalyst was not pyrolysed under 5% of H_2/N_2 again.]

Catalyst / at%	Pyridinic N	M-N	Prrolyic N	Graphitic N	N oxide
(Zn)-N/C^U	48.03	3.68	33.71	9.89	4.7
(Zn)-N/C	48.56	7.3	29.53	9.76	4.85
Fe-N/C	44.82	13.75	28.38	9.24	3.8
Co-N/C	46.84	16.81	23.77	7.61	4.97
Ni-N/C	51.06	17.73	20.29	6.38	4.54
Sn-N/C	46.44	14.37	25.3	9.5	4.39
Sb-N/C^U	55.95	10.4	21.59	7.54	4.52
Bi-N/C	53.86	13.29	24.46	4.79	3.59

Sb-N/C^U and N/C^U are unactivated catalysts which did not undergo a second pyrolysis step.

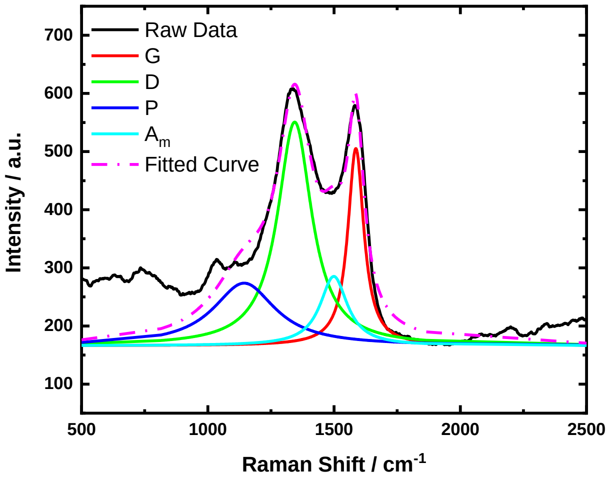

Fig S16. ¦ Fitting of Raman spectrum of Fe-N/C catalyst.

Graphitic peak(G) is shown at 1573-1600 cm^{-1} and disorder peak(D) is shown at 1340-1356 cm^{-1}.7 Those two major peaks are due to different vibration mode of graphene (E_{2g} and A_{1g}). The other two minor peak are at 1451- 1531 cm^{-1} and 1144-1190 cm^{-1} and they are amorphous carbon (Am) and pentacene (P) peaks.[1] According to the integrated intensity ratio, the average graphene domain size could be calculated. Furthermore, the percentage of edges, basal and buried site density is calculated and they are shown in Table S5.

Table S5 ¦ Estimate the different sites within Fe-N/C and the percentages.

Analysis based on the Raman of Fe-N/C are published by Mehmood *et al.* [1] Since the catalysts in paper[1] are prepared by the same method as in this work, the estimated values of edge, basal and buried sites are expected to be similar. Errors are given in parentheses, e.g. 6.0(2) means 5.8-6.2

**Site**	**Site density / sites g^{-1}**	**Site density / %**
Edge	$6(2)\times10^{19}$	8(3)
Basal	$6.0(2)\times10^{20}$	79(3)
Buried	$1.0(2)\times10^{20}$	13(3)
Total	$7.6(2)\times10^{20}$	100.0

For the determination of site density as a function of specific surface area and wt%(Fe), a simple geometrical model was used, in which we assume the catalyst is composed of iron sites randomly embedded within graphene like sheets of semi-infinite dimensions. The graphene like sheets form multi-layer structures which contain both surface iron sites and internal iron sites associated with "buried" iron. The total number of sites is then the accessible number on the surface.

Raman spectra are used to determine the lateral diamension (L_a) of the graphene sheet. As we know the total number of iron sites from the at% loading (7.55 x10^{20} sites g^{-1}), and we also know the total number of surface sites 6.54 x 10^{20} sites g^{-1} and the percentage of surface sites that are basal sites (91.1%) we can classify the iron sites into the three different sites – basal, edge and buried, where the latter is associated with iron not on the surface of the catalyst. Errors are calculated based upon propagation of the uncertainty of $L_{a.}$

The detailed calculation methods are shown in the Supporting Information of paper published by Mehmood *et al.* [1]

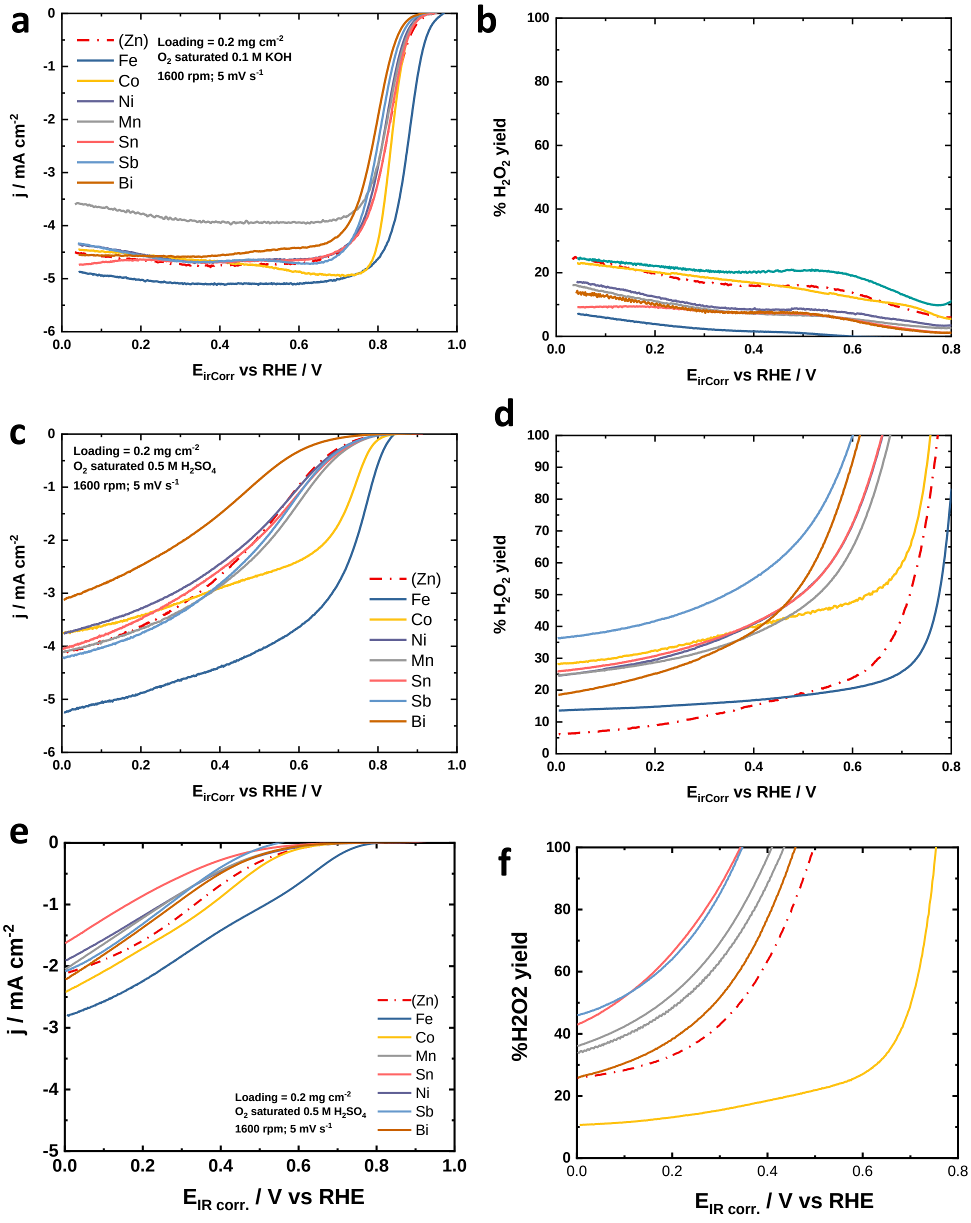

Fig S17. ¦ **Oxygen Reduction Reaction (ORR) performances of M-N/Cs** in (a) 0.1 M KOH; (c) 0.5 M H_2SO_4; (e) M-N/C^{U}s in 0.5 M H_2SO_4; %H_2O_2 yield of M-N/Cs in (b) 0.1 M KOH; (d) 0.5 M H_2SO_4; (f) M-N/C^{U}s in 0.5 M H_2SO_4. All measurements were done with 1,600 rpm rotation and scanning rate 5 mVs^{-1}.

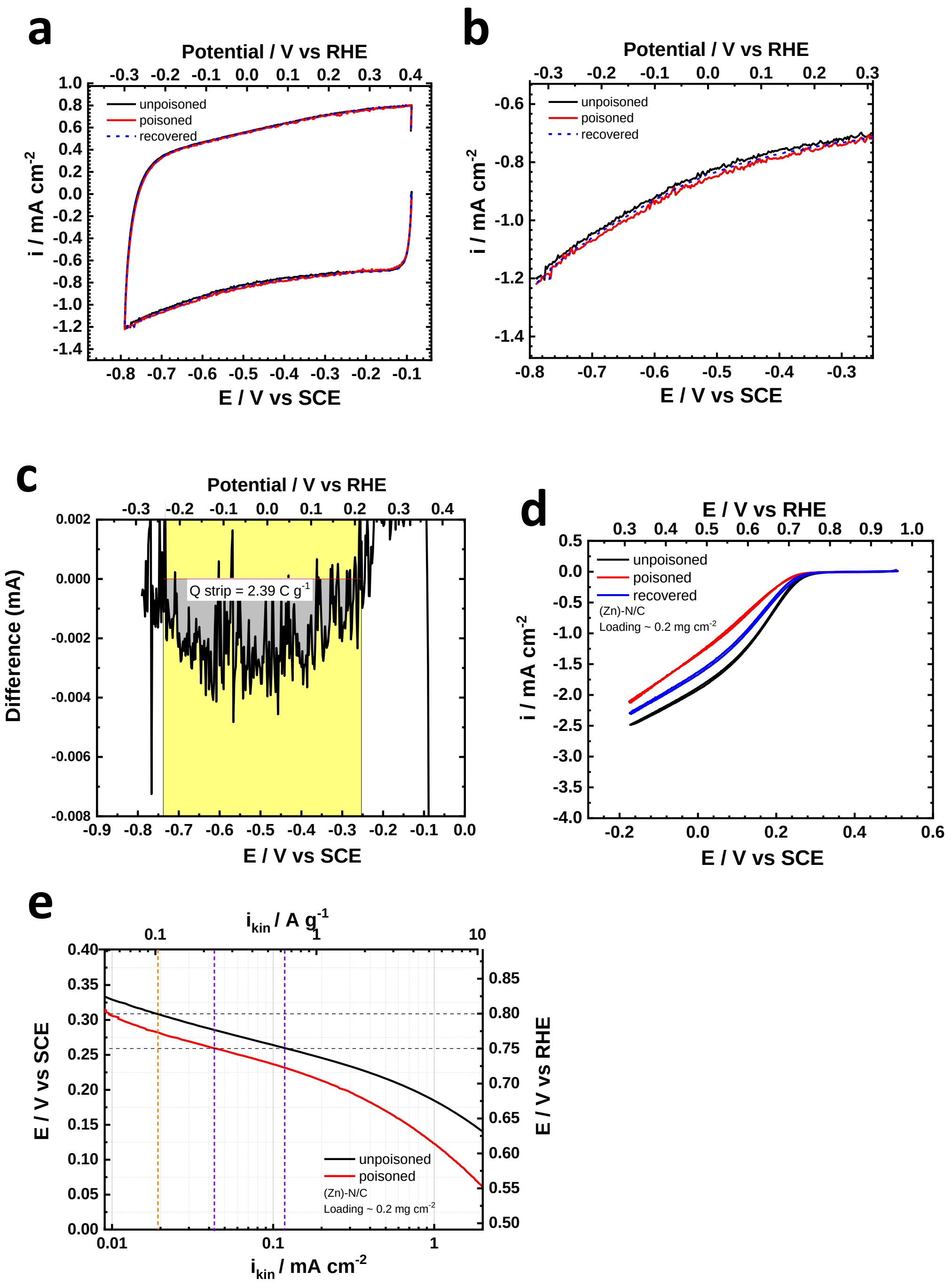

Fig S18. ¦ **Nitrite stripping of (Zn)-N/C** (a) N_2 baseline CVs; (b) zoom-in image of N_2 baseline CVs; (c) stripping charge calculated based on the N_2 baseline CVs; (d) ORR polarisation curves; (e) shifts in kinetic current before and after poisoning. Black line: unpoisoned catalyst; Red line: poisoned catalyst; blue line: recovered catalyst.

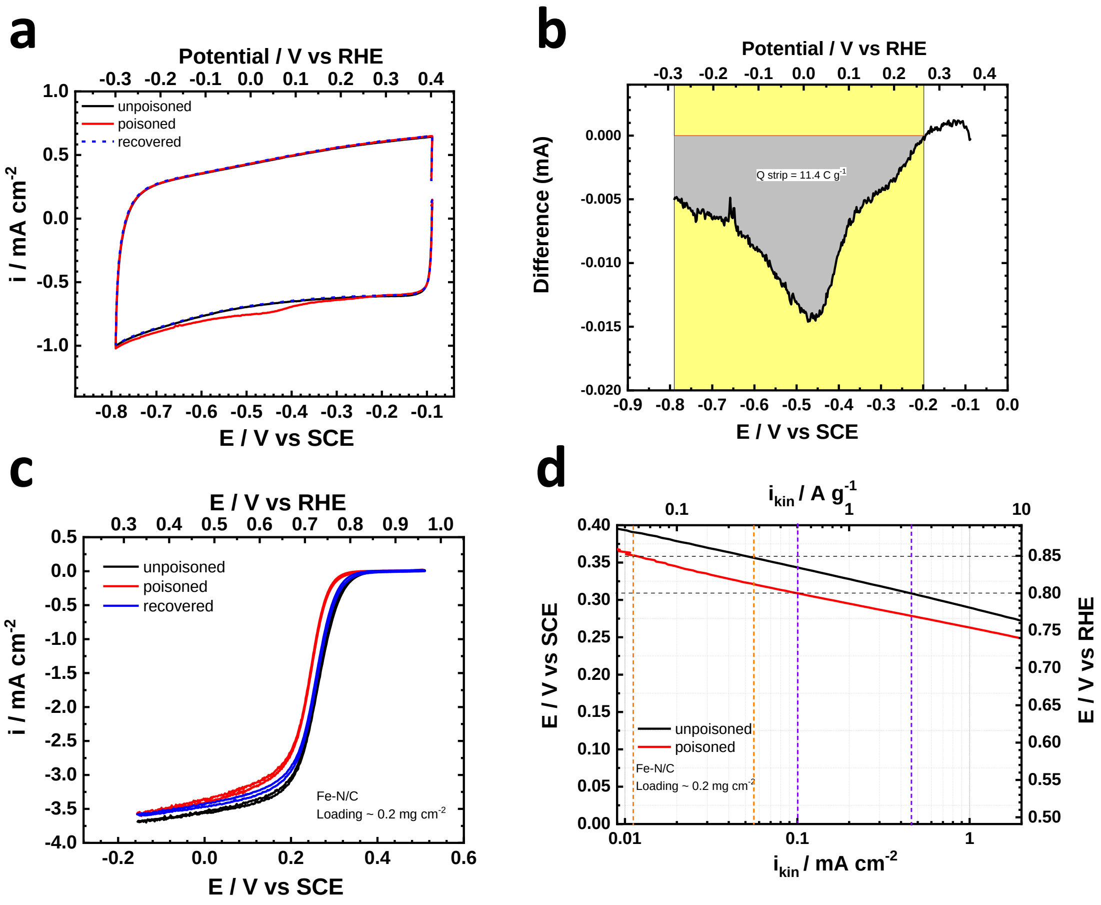

Fig S19. ¦ Nitrite stripping of Fe-N/C (a) N_2 baseline CVs; (b) stripping charge calculated based on the N_2 baseline CVs; (c) ORR polarisation curves; (d) shifts in kinetic current before and after poisoning. Black line: unpoisoned catalyst; Red line: poisoned catalyst; blue line: recovered catalyst.

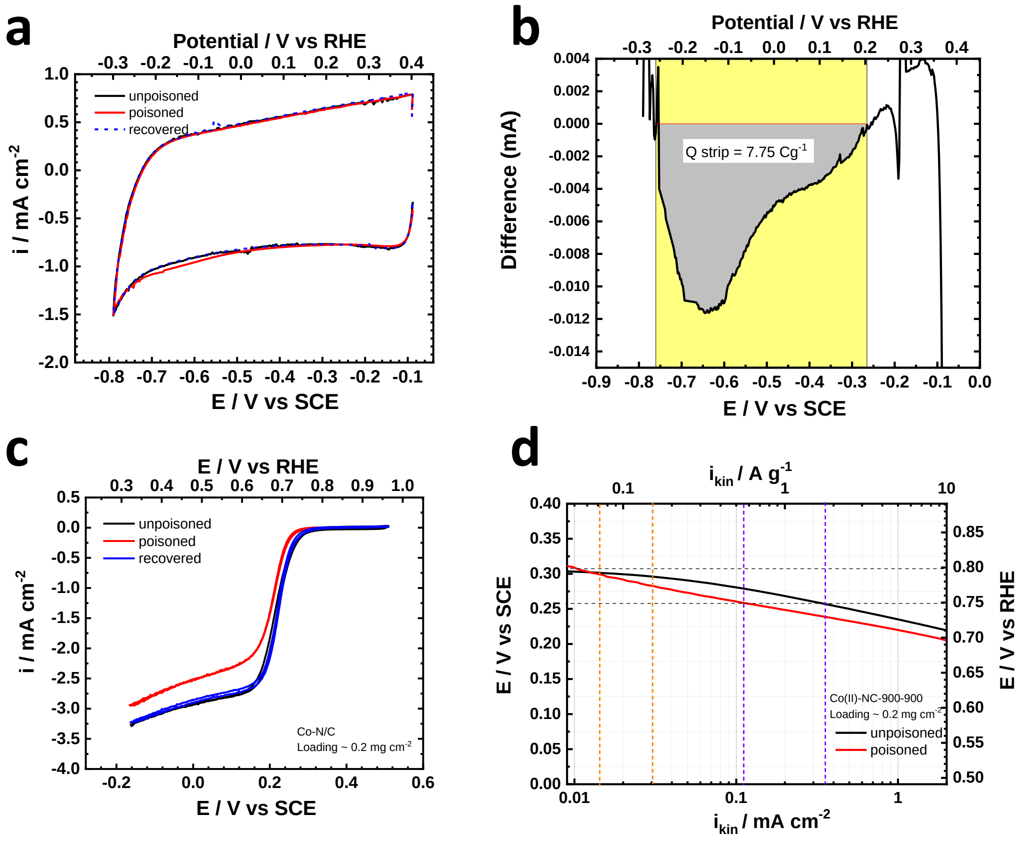

Fig S20. ¦ **Nitrite stripping of Co-N/C** (a) N_2 baseline CVs; (b) stripping charge calculated based on the N_2 baseline CVs; (c) ORR polarisation curves; (d) shifts in kinetic current before and after poisoning. Black line: unpoisoned catalyst; Red line: poisoned catalyst; blue line: recovered catalyst.

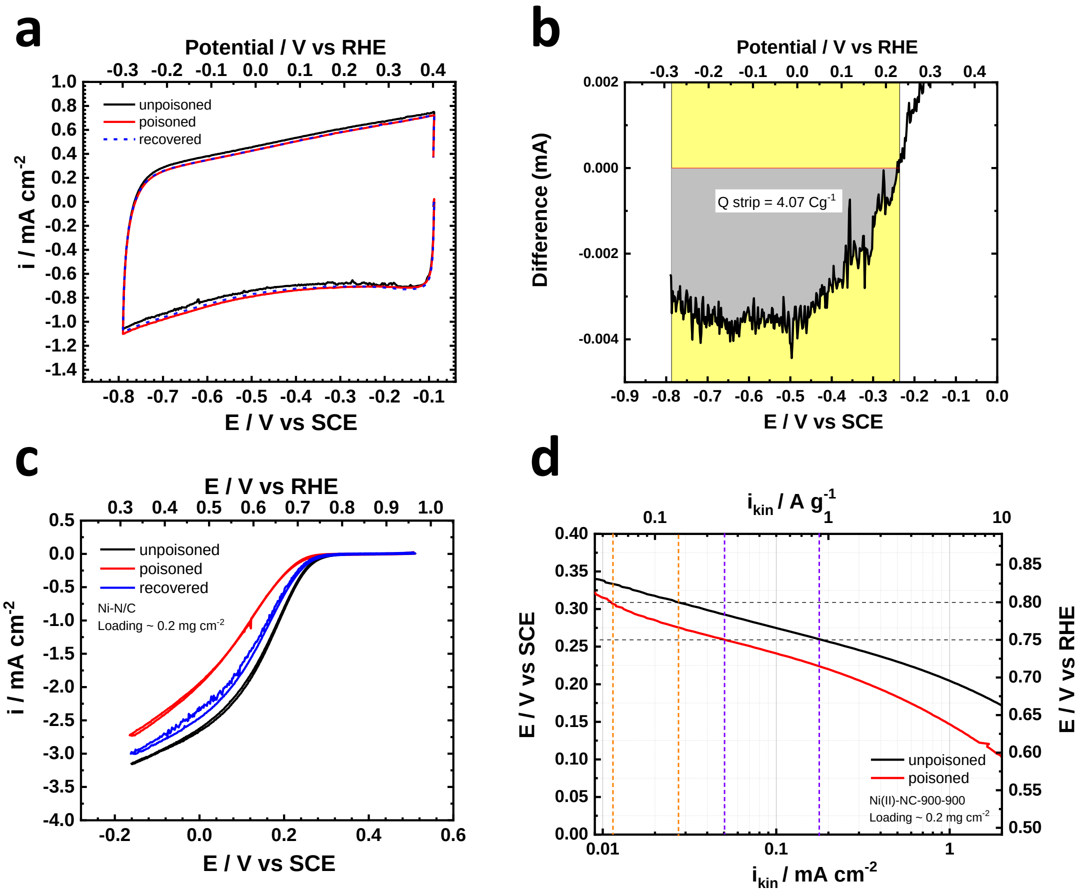

Fig S21. ¦ Nitrite stripping of Ni-N/C (a) N_2 baseline CVs; (b) stripping charge calculated based on the N_2 baseline CVs; (c) ORR polarisation curves; (d) shifts in kinetic current before and after poisoning. Black line: unpoisoned catalyst; Red line: poisoned catalyst; blue line: recovered catalyst.

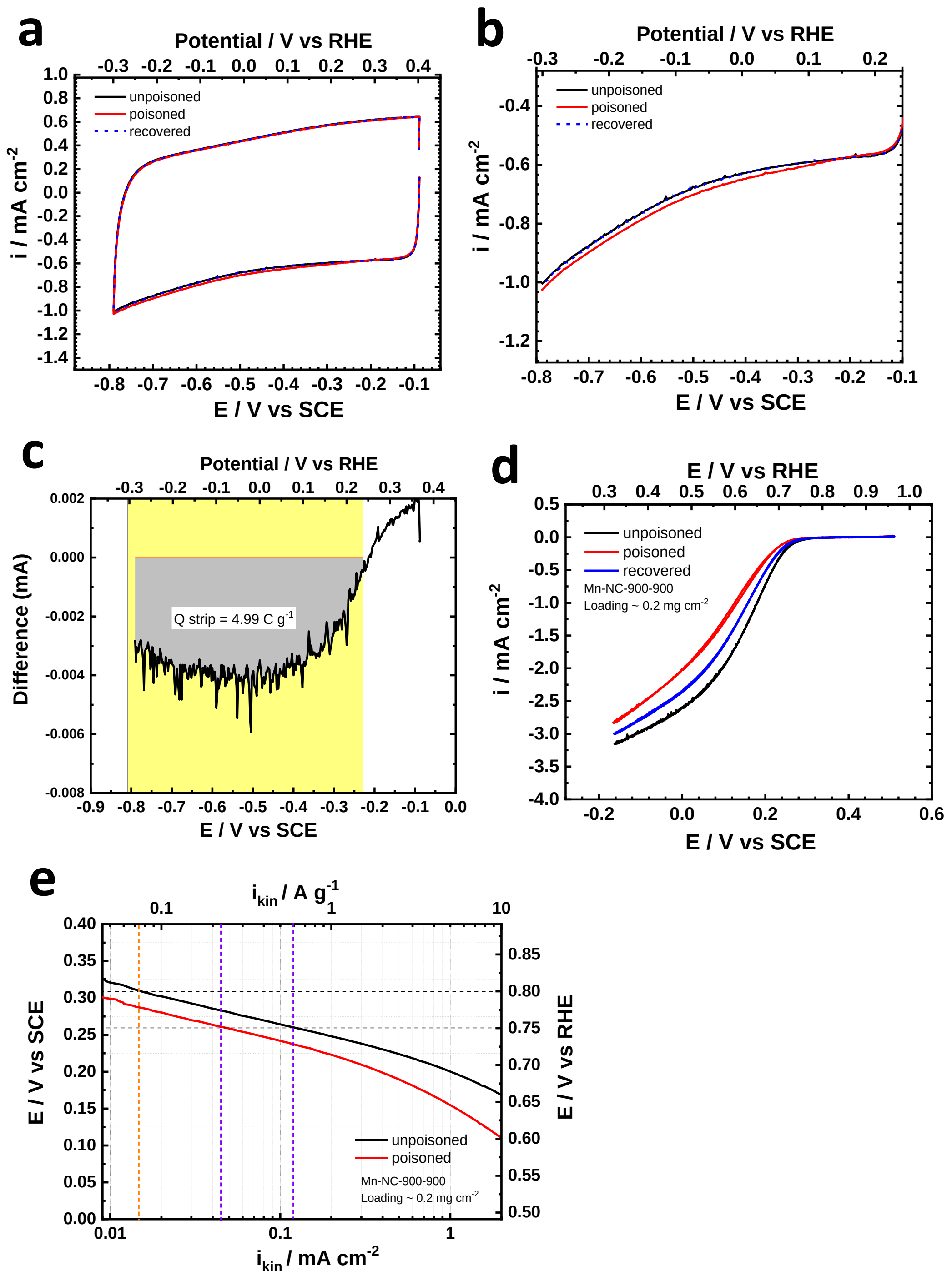

Fig S22. ¦ Nitrite stripping of Mn-N/C (a) N_2 baseline CVs; (b) zoom-in image of N_2 baseline CVs; (c) stripping charge calculated based on the N_2 baseline CVs; (d) ORR polarisation curves; (e) shifts in kinetic current before and after poisoning. Black line: unpoisoned catalyst; Red line: poisoned catalyst; blue line: recovered catalyst.

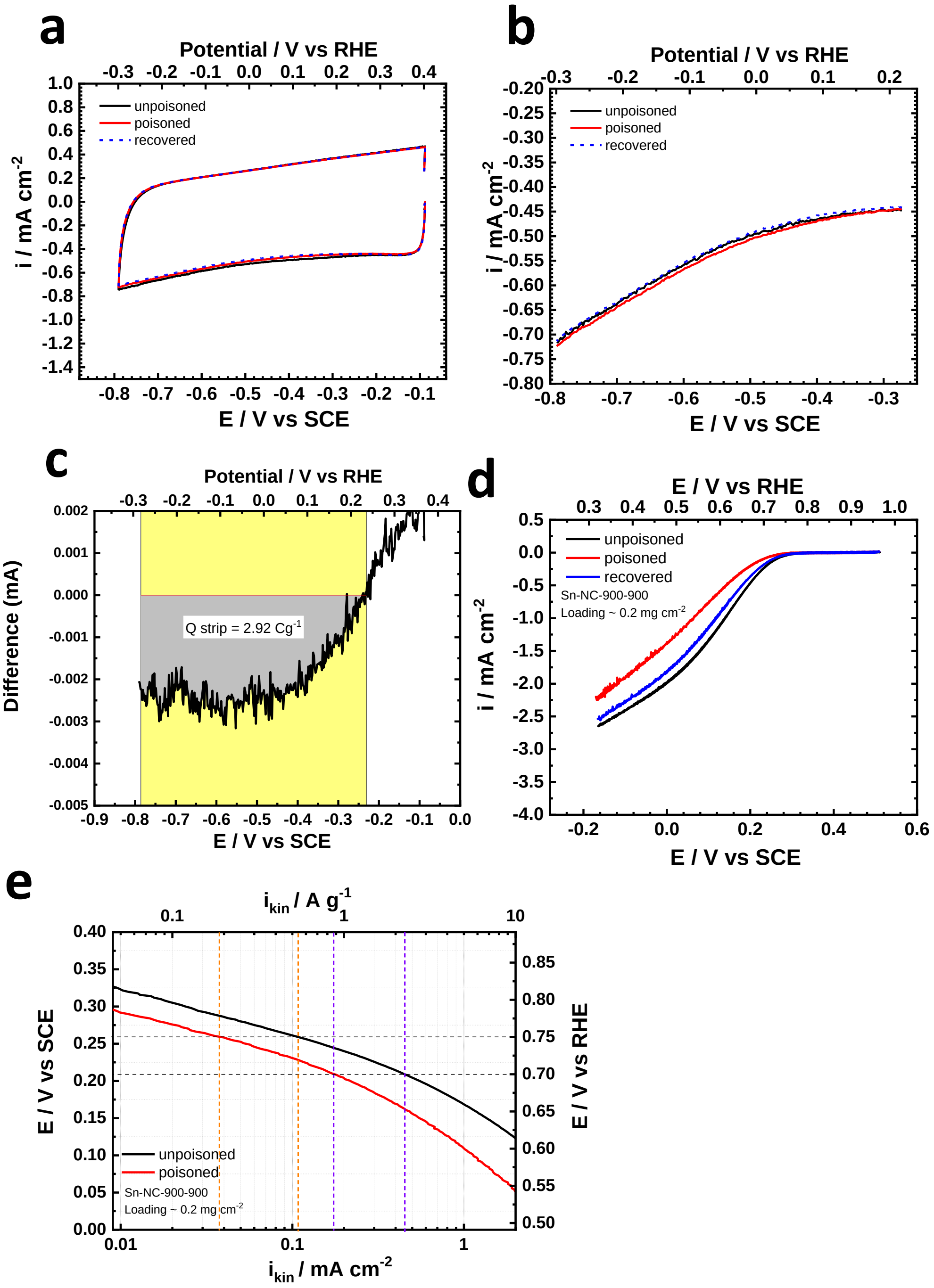

Fig S23. ¦ **Nitrite stripping of Sn-N/C** (a) N_2 baseline CVs; (b) zoom-in image of N_2 baseline CVs; (c) stripping charge calculated based on the N_2 baseline CVs; (d) ORR polarisation curves; (e) shifts in kinetic current before and after poisoning. Black line: unpoisoned catalyst; Red line: poisoned catalyst; blue line: recovered catalyst.

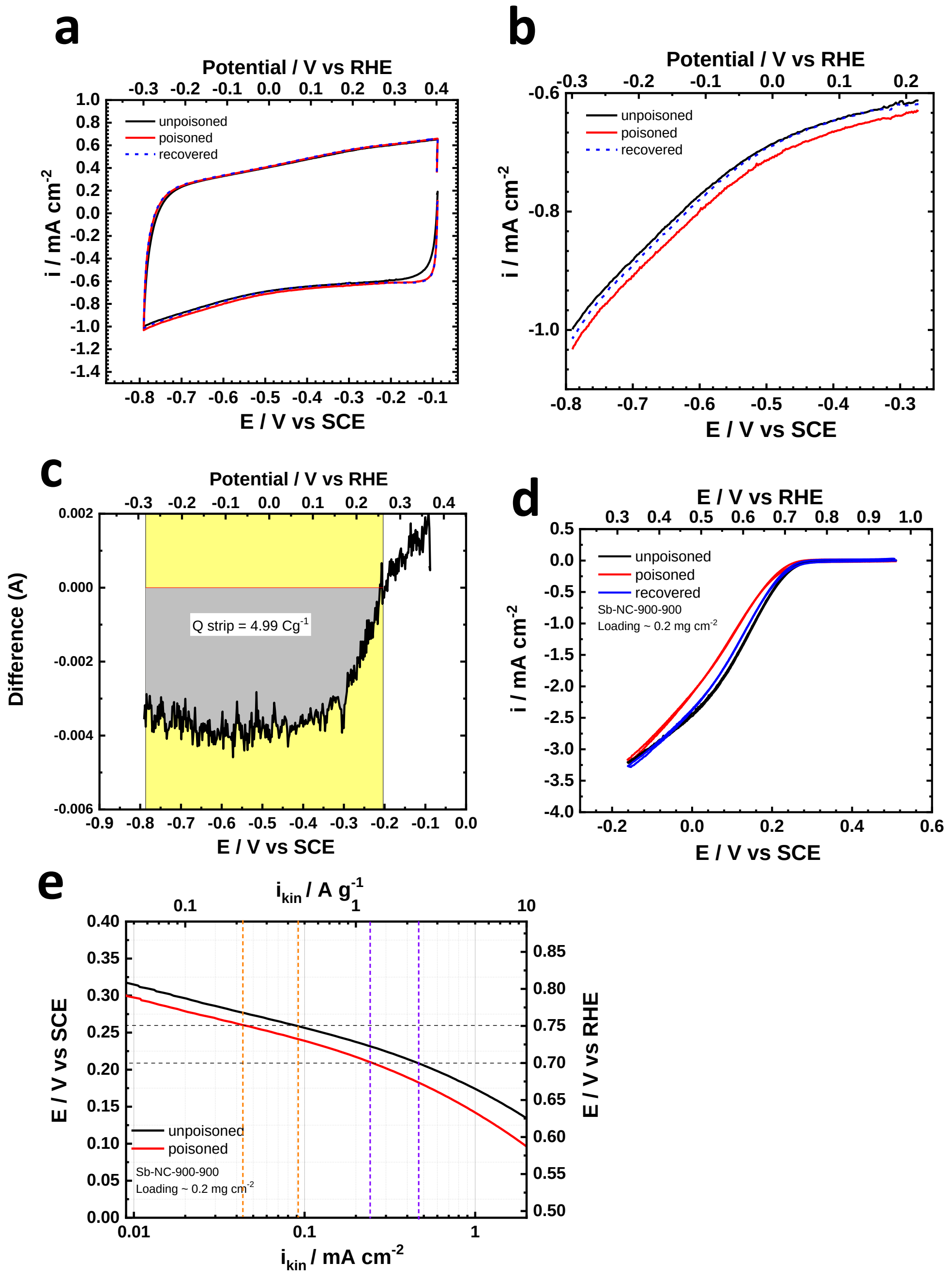

Fig S24. ¦ **Nitrite stripping of Sb-N/C** (a) N_2 baseline CVs; (b) zoom-in image of N_2 baseline CVs; (c) stripping charge calculated based on the N_2 baseline CVs; (d) ORR polarisation curves; (e) shifts in kinetic current before and after poisoning. Black line: unpoisoned catalyst; Red line: poisoned catalyst; blue line: recovered catalyst.

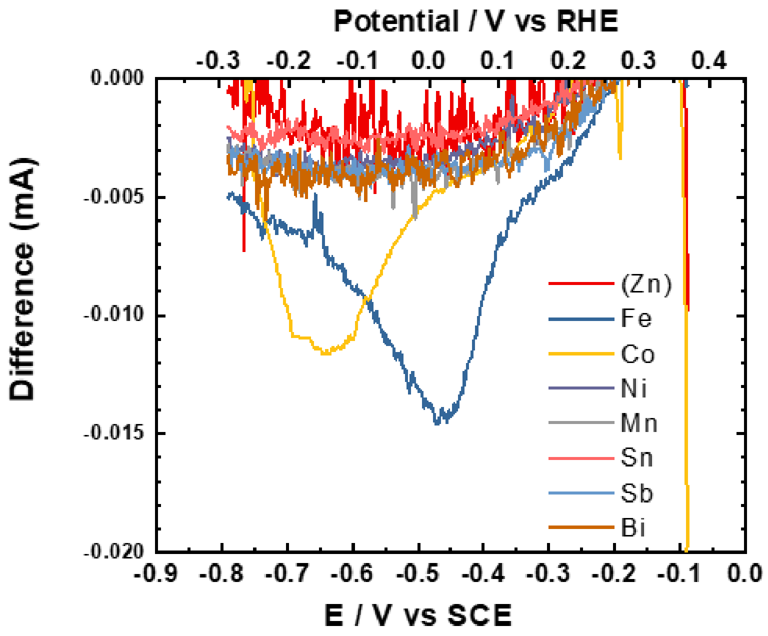

Fig S25. ¦ Difference of nitrite stripping currents between poisoned and unpoisoned catalysts for all tested M-N/Cs.

Only for Fe-N/C and Co-N/C, there are distinct NO stripping peaks and the peak potentials are changing depending on nature of metal sites. Rest of the M-N/Cs have very similar NO stripping pattern without any well-defined reducing peak, which suggests the NO interacts with the framework N/C sites instead of M-N_x sites.

Table S6 ¦ The site density and TOF values of individual framework and metal sites.

Sites	Site density/ 10^{18} sites g^{-1}	$TOF_{pH\ 0.3}$			$TOF_{pH\ 5.2}$			$TOF_{pH\ 13.0}$		
		0.70 V	0.75 V	0.80 V	0.70 V	0.75 V	0.80 V	0.70 V	0.75 V	0.80 V
**Framework**	4.88	1.98	0.70	0.12	3.30	0.72	0.08	614.2	186.6	45.11
**$Fe-N_4$**	9.35	21.5	9.81	2.14	49.5	13.4	1.31	541.9*	322.8*	171.8
**$Co-N_4$**	4.79	19.9	5.93	0.65	20.7	2.65	0.35	1530*	3033*	408.2

* This value is extrapolated from the Tafel slope as shown in the main manuscript. As the Co-N/C and Fe-N/C have already reached the limiting currents at 0.80 V, therefore, the value looks extremely high especially for the Co-N/C.

The TOFs reported in this table are calculated from the activities deconvoluted by different sites and their site densities, instead of total TOF. This method not only provides information about the site densities and the activities of different active centres (framework N/C sites and $M-N_4$ sites), but also allows to predict the activity of catalysts with different site densities. Moreover, if the site density of $M-N_4$ is quite small, then the activity of catalyst would be similar with the activity contributed by the framework N/C.

References

(1) Mehmood, A.; Gong, M.; Jaouen, F.; Roy, A.; Zitolo, A.; Khan, A.; Sougrati, M.-T.; Primbs, M.; Bonastre, A. M.; Fongalland, D.; et al. High loading of single atomic iron sites in Fe–NC oxygen reduction catalysts for proton exchange membrane fuel cells. *Nature Catalysis* **2022**, *5* (4), 311-323. DOI: 10.1038/s41929-022-00772-9.
